# A Synergistic Three-Phase, Triple-Conducting Air Electrode for Reversible Proton-Conducting Solid Oxide Cells

**DOI:** 10.1021/acsenergylett.3c01251

**Published:** 2023-09-01

**Authors:** Weilin Zhang, Yucun Zhou, Xueyu Hu, Yong Ding, Jun Gao, Zheyu Luo, Tongtong Li, Nicholas Kane, Xiao-Ying Yu, Tanguy Terlier, Meilin Liu

**Affiliations:** † School of Materials Science and Engineering, Georgia Institute of Technology, Atlanta, Georgia 30332-0245, United States; ‡ Energy and Environment Directorate, Pacific Northwest National Laboratory, Richland, Washington 99354, United States; § Energy Materials and Surface Sciences Unit, Okinawa Institute of Science and Technology Graduate University, 1919-1 Tancha, Onna-son, Kunigami-gun, Okinawa 904-0495, Japan; ∥ Materials Science and Technology Division, Oak Ridge National Laboratory, Oak Ridge, Tennessee 99354, United States; ⊥ Shared Equipment Authority, SIMS Laboratory, Rice University, Houston, Texas 77005, United States

## Abstract

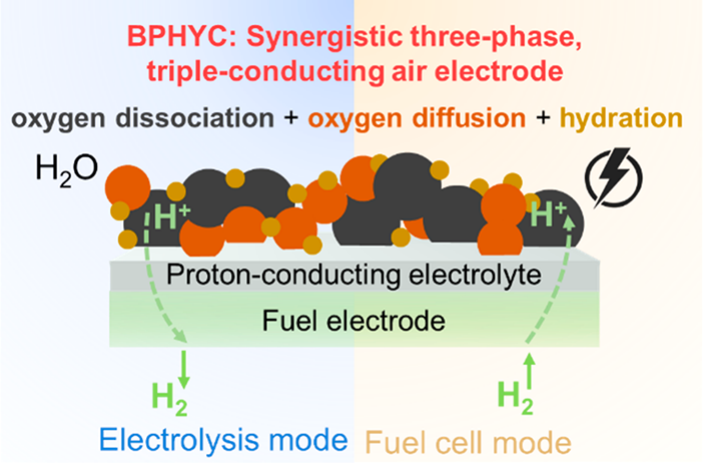

Reversible proton-conducting solid oxide cells (R-PSOCs) have the potential to be the most efficient and cost-effective electrochemical device for energy storage and conversion. A breakthrough in air electrode material development is vital to minimizing the energy loss and degradation of R-PSOCs. Here we report a class of triple-conducting air electrode materials by judiciously doping transition- and rare-earth metal ions into a proton-conducting electrolyte material, which demonstrate outstanding activity and durability for R-PSOC applications. The optimized composition Ba_0.9_Pr_0.1_Hf_0.1_Y_0.1_Co_0.8_O_3−δ_ (BPHYC) consists of three phases, which have a synergistic effect on enhancing the performance, as revealed from electrochemical analysis and theoretical calculations. When applied to R-PSOCs operated at 600 °C, a peak power density of 1.37 W cm^–2^ is demonstrated in the fuel cell mode, and a current density of 2.40 A cm^–2^ is achieved at a cell voltage of 1.3 V in the water electrolysis mode under stable operation for hundreds of hours.

The growing energy consumption and environmental concerns have stimulated the development of efficient and reliable technologies for the storage and conversion of renewable energies (e.g., wind and solar) due to their intermittent nature. Reversible solid oxide electrochemical cells (R-SOCs) are highly efficient electrochemical devices that can operate in both the fuel cell mode to generate electricity and the electrolysis mode to produce hydrogen or other cleaner fuels (by electrolyzing water and carbon dioxide).^1,2^ Compared to other energy storage and conversion technologies, R-SOCs have the advantages of high efficiency, low cost, and good durability.^3^

Compared to R-SOCs based on oxygen-ion-conducting electrolytes,^4,5^ the R-SOCs based on proton-conducting electrolytes (or reversible proton-conducting solid oxide cells, R-PSOCs) have the potential to be more efficient and durable. First, the activation energy of proton conduction is much lower than that of the oxygen ion. Thus, R-PSOCs have the potential to operate more efficiently at lower temperatures (∼500 °C) than oxygen-ion-conducting R-SOCs (>700 °C),^6^ implying that much less expensive materials may be used to reduce the cost. Second, water is produced on the air electrode side of R-PSOCs in the fuel cell mode, potentially improving fuel utilization.^7^ Third, dry hydrogen is produced on the fuel electrode side of R-PSOCs in the electrolysis mode, simplifying the system and reducing system costs while eliminating the concern of Ni oxidation due to exposure to steam.^8,9^ Currently, the development of R-PSOCs is hindered by poor electrochemical activity and stability of the air electrode material.^10^ Even though some mixed ionic and electronic conductors (MIECs) including La_0.6_Sr_0.4_Co_0.2_Fe_0.8_O_3−δ_ (LSCF) and Ba_0.5_Sr_0.5_Co_0.8_Fe_0.2_O_3−δ_ (BSCF) have been used as the air electrode material, they are not suitable for R-PSOC application due to the poor activity at low temperatures, limited proton conductivity, and instability under high concentrations of steam when operating under the electrolysis mode.^11,12^ An effective way to design triple-conducting air electrode materials is by heavily doping transition-metal ions into proton-conducting electrolyte materials in order to improve the electronic conductivity as well as the catalytic activity.^6^ Recently, nanocomposite materials also demonstrated great potential as air electrodes for R-PSOCs.^13,14^

In this study, a series of triple-conducting air electrode materials were designed, synthesized, and optimized by rationally doping transition- and rare-earth metal ions into BaHf_0.8_Y_0.2_O_3−δ_, which was recently developed as an active and stable electrolyte material for R-PSOCs.^15^ The optimized BPHYC air electrode demonstrated outstanding electrochemical performance on R-PSOCs, achieving a peak power density of 1.37 W cm^–2^ in the fuel cell mode and a current density of 2.40 A cm^–2^ at 1.3 V in the electrolysis mode at 600 °C (Figure 1). A detailed analysis showed the coexistence of three different phases in BPHYC, and the synergistic effect of these phases was confirmed by electrochemical measurements and theoretical calculations. More importantly, R-PSOCs with the BPHYC air electrode demonstrated good stability under different operating conditions for over 500 h at intermediate temperatures. The study revealed the function of each phase in the composite electrode, providing important insight for the rational design of more efficient electrodes. This work also demonstrated the potential to fabricate a high-performance air electrode via a simple and scalable SSR method, which is cost-effective for mass production of high-performance electrode materials.

**Figure 1:**
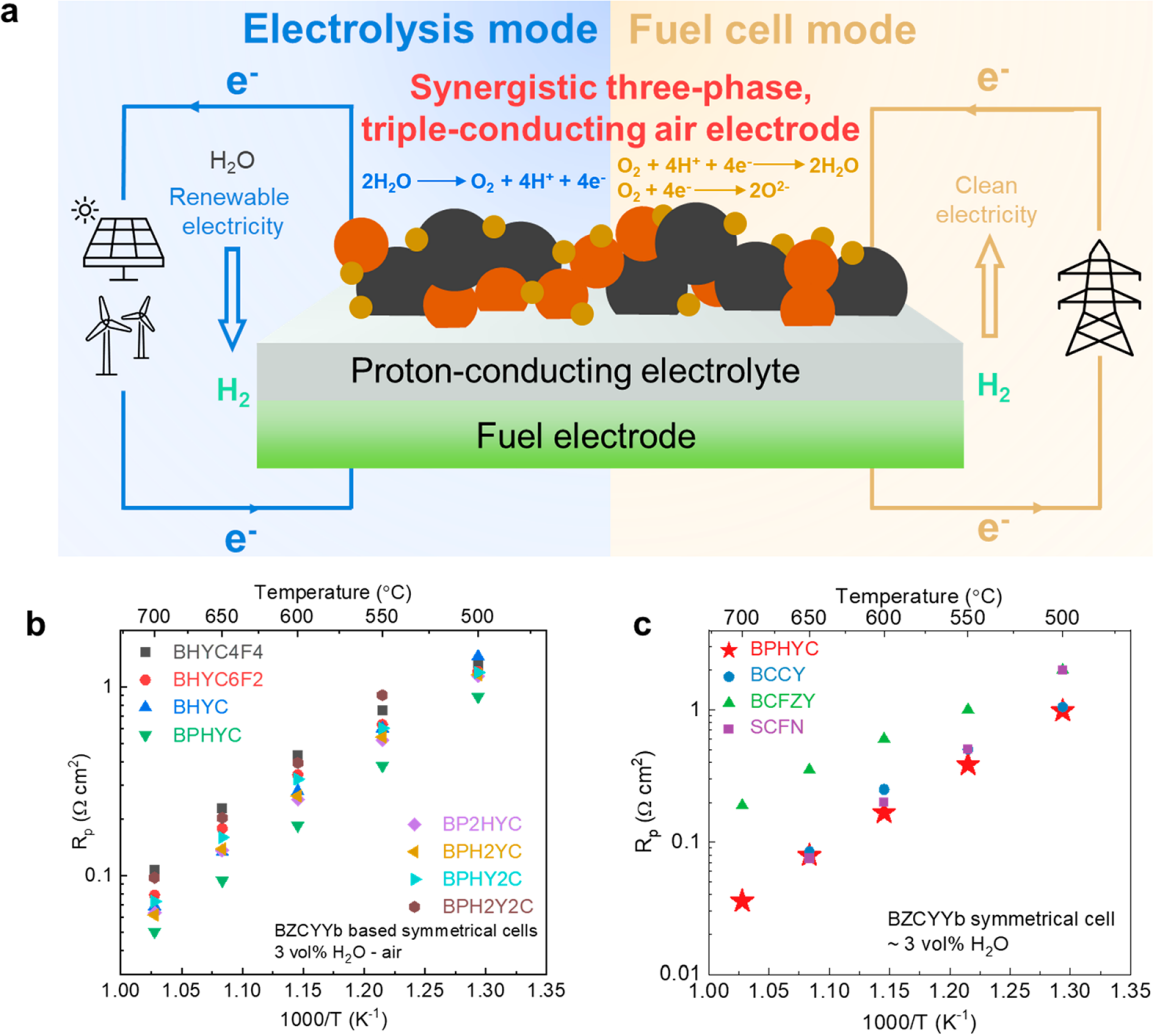
(a) A synergistic three-phase, triple-conducting air electrode for R-PSOCs. (b) Interfacial polarization resistance (*R*_p_) of the air electrode candidates as a function of temperature on BaZr_0.1_Ce_0.7_Y_0.1_Yb_0.1_O_3-δ_ (BZCYYb) symmetrical cells. The detailed composition of each candidate is given in Table S1. (c) Electrochemical performance comparison between BPHYC and other state-of-the-art triple-conducting air electrode materials. Abbreviations: BCCY, BaCo_0.7_ (Ce_0.8_Y_0.2_)_0.3_O_3−δ_;^13^ BCFZY, BaCo_0.4_Fe_0.4_Zr_0.1_Y_0.1_O_3−δ_;^6^ SCFN, Sr_0.9_Ce_0.1_Fe_0.8_Ni_0.2_O_3−δ_.^14^

To explore the BaHf_0.8_Y_0.2_O_3−δ_-based material system, Co, Fe, and Pr were doped into BaHf_0.8_Y_0.2_O_3−δ_ to synthesize the air electrode powder via a solid-state reaction method (Figure S1). The ratio of each element was controlled by the amount of precursor oxide used (Table S1). The X-ray diffraction (XRD) patterns of the candidate materials are shown in Figure S2. The electrochemical performance of the different air electrodes was characterized on BZCYYb-based symmetrical cells in air with 3 vol % H_2_O. Figure 1b compares the *R*_p_ of each material from 500 to 700 °C. Among all of the material candidates, BPHYC shows the lowest R_p_, surpassing other triple-conducting air electrode materials reported to date, especially at temperatures below 600 °C (Figure 1c), making it a potential air electrode candidate for R-PSOC applications.

The XRD pattern of the BPHYC air electrode is shown in Figure 2a. Three different phases were observed. One cubic perovskite phase corresponds to Y-doped BaCoO_3−δ_ (BYC, phase A), one double-perovskite phase corresponds to PrBaCo_2_O_5+δ_ (PBC, phase B), and one cubic perovskite phase corresponds to Y-doped BaHfO_3−δ_ (BHY, phase C). No extra peaks were detected. Detailed information about the composition and lattice parameters of each phase is given in Table S2. Figure 2b–d shows the high-resolution scanning transmission electron microscopy (HR-STEM) images, fast Fourier transform (FFT) pattern, and transmission electron microscopy (TEM) images of each phase in BPHYC. The lattice parameters calculated from the HR-STEM images correspond to the XRD refinement results (Table S2). Because BHY is more resistant to particle coarsening than Co-rich materials during the high-temperature firing process, the particle size of BHY is much smaller (∼30 nm in Figure 2d) than those of BYC (∼1 um in Figure 2b) and PBC (∼500 nm in Figure 2c). These results further confirm the existence of three phases in BPHYC. XRD patterns of BPHYC after firing at different temperatures are shown in Figure S3. Firing at 1050 °C for 12 h successfully creates the three phases.

**Figure 2:**
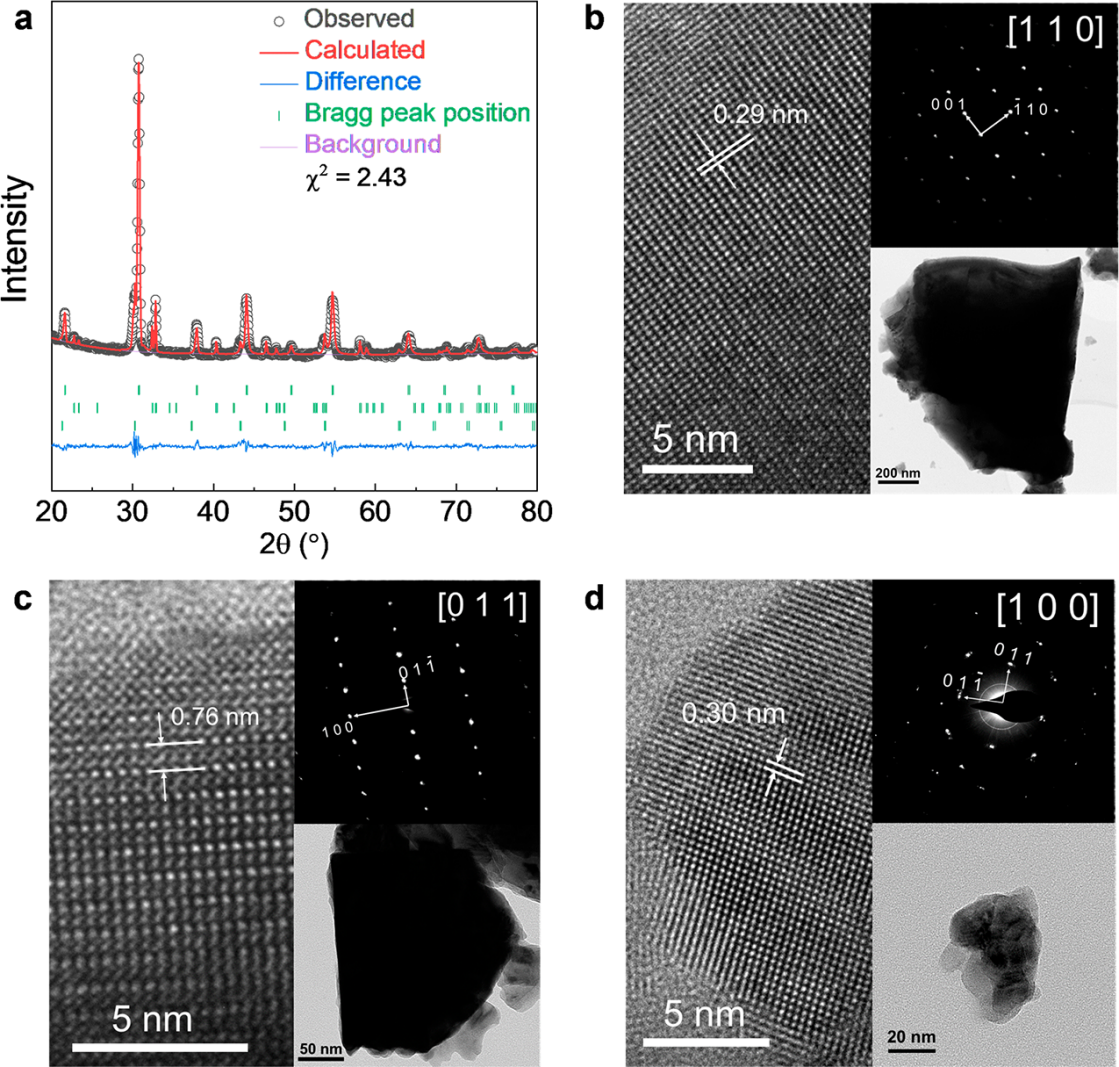
Structure of BPHYC. (a) XRD pattern of BPHYC, in which three phases were identified. (b) HR-STEM image of BYC. Inset: FFT pattern along the [110] zone axis of BYC and the TEM image of the BYC particle. (c) HR-STEM image of PBC. Inset: FFT pattern along the [001] zone axis of PBC and the TEM image of the PBC particle. (d) HR-STEM image of BHY. Inset: FFT pattern along the [100] zone axis of BHY and the TEM image of the BHY particle.

The effect of each phase on the oxygen reduction reaction (ORR) activity in BPHYC was then investigated. Based on previous studies, doped BaCoO_3−δ_ and PBC have been widely applied on oxygen-ion-conducting solid oxide fuel cells (O-SOFCs) as cathode and catalyst materials with excellent ORR activity.^7,16−19^ BHY-based materials are stable electrolyte materials with good protonic conductivity, especially at low temperatures.^15^ Thus, we propose that these three phases may have a synergistic effect on the ORR activity when applied to proton-conducting electrolytes. To validate this hypothesis, a mixture of BYC and PBC (BPYC), at the same BYC to PBC ratio in BPHYC, was synthesized by the same SSR method (Figures S1 and S4). XRD refinement results (Figure S5 and Table S3) confirm that the BYC to PBC ratio in the BPHYC electrode is comparable to that in the BPYC electrode. BET analysis (Figure S6) also shows that BPHYC and BPYC have similar surface areas, indicating a similar contribution of the microstructure and morphology to the electrochemical performance. Single-phase BYC and PBC were also synthesized using the same method (Figure S4). To focus on the oxygen-ion conduction and to eliminate the contribution of proton conduction, Sm_0.2_Ce_0.8_O_1.9_ (SDC)-based symmetrical cells with BYC, PBC, BPYC, and BPHYC air electrodes were fabricated. Figure 3a compares the *R*_p_ values of these air electrode materials in dry air. By adding the BYC (phase A) and PBC (phase B) together, the *R*_p_ value of BPYC (A+B) was smaller than those of BYC and PBC, indicating that BYC and PBC have a synergistic effect on the kinetics of the ORR. Further adding BHY (phase C) to BPYC does not have an obvious effect on the electrochemical performance when applied to an SDC electrolyte. To gain a deeper understanding of the effect of BYC and PBC on the ORR, we performed a distribution of relaxation time (DRT) analysis on these symmetrical cells. Figure S7 shows the electrochemical impedance spectroscopy (EIS) plots of BYC, PBC, and BPYC symmetrical cells as a function of the oxygen partial pressure. The total *R*_p_ decreases as the oxygen partial pressure increases from 0.2 to 1.0 atm. DRTTOOLs was used to analyze the complex impedance spectra.^20^Figure 3b shows the DRT plot of BYC, PBC, and BPYC at an oxygen partial pressure of 0.2 atm. Three distinct peaks were clearly shown at high frequency (HF), intermediate frequency (IF), and low frequency (LF). Compared to BYC, the LF peak intensity of PBC is lower, and the IF peak intensity of PBC is higher. The HF peaks of BYC and PBC are almost the same. The two-phase BPYC takes advantage of both the BYC at IF and PBC at LF, as shown in Figure 3b, resulting in an overall reduced *R*_p_. The integral area under each peak represents the total impedance of the process corresponding to a certain frequency. The general dependence of *R*_p_ on *P*_O_2__ can be written by the equation *R*_p_ = *k*(*P*_O_2__)^−*n*^. Figure 3c summarizes the *R*_p_ values of BPYC at different frequencies as a function of the oxygen partial pressure. The *R*_p_ value of the HF process is almost independent of the oxygen partial pressure, which means the HF process is likely associated with the transport of oxygen species through the electrolyte/electrode interface.^19^ At IF, the *n* value is close to 0.25, indicating that the IF process relates to the diffusion of intermediate oxygen species.^21^ At LF, the *n* value is close to 0.5, indicating that the LF process relates to the oxygen dissociation process.^21,22^ Based on the detailed DRT analysis, PBC shows higher activity on the oxygen dissociation process (at LF), and BYC shows better activity on the oxygen diffusion (at IF). BPYC takes advantage of faster oxygen diffusion from the BYC phase and faster oxygen dissociation from the PBC phase, achieving an improved overall ORR activity. DFT-based calculations were performed to have a deeper understanding of the synergistic effect of BYC and PBC. First, slab models of BYC, PBC, and BPYC were optimized (Figures S8–S10). All possible oxygen vacancy sites at the BYC, PBC, and BPYC surfaces were considered (Figures S11 and S12), and the corresponding oxygen vacancy formation energy were calculated as shown in Figure 3d. For the next step, the oxygen adsorption process at the most favorable sites were calculated (site 4 for BYC, site 8 for PBC, and site 3 for BPYC as shown in Figure 3e). Compared to BYC and PBC, a lower oxygen adsorption energy is shown for BPYC. Then we simulated the oxygen dissociation process on these three materials (Figures S8, S9, and S13), which is the rate-limiting step in the ORR.^23^ The energy change during the oxygen adsorption and dissociation processes is shown in Figure 3f. The combination of the BYC and PBC phases resulted in a lower activation energy for the oxygen dissociation process, which is consistent with the lowest *R*_p_ of BPYC from the experimental results (Figure 3a). The Bader charge result in Figure S14 indicates that the oxygen dissociation process in our theoretical calculation also includes the charge transfer process in the DRT analysis.

**Figure 3:**
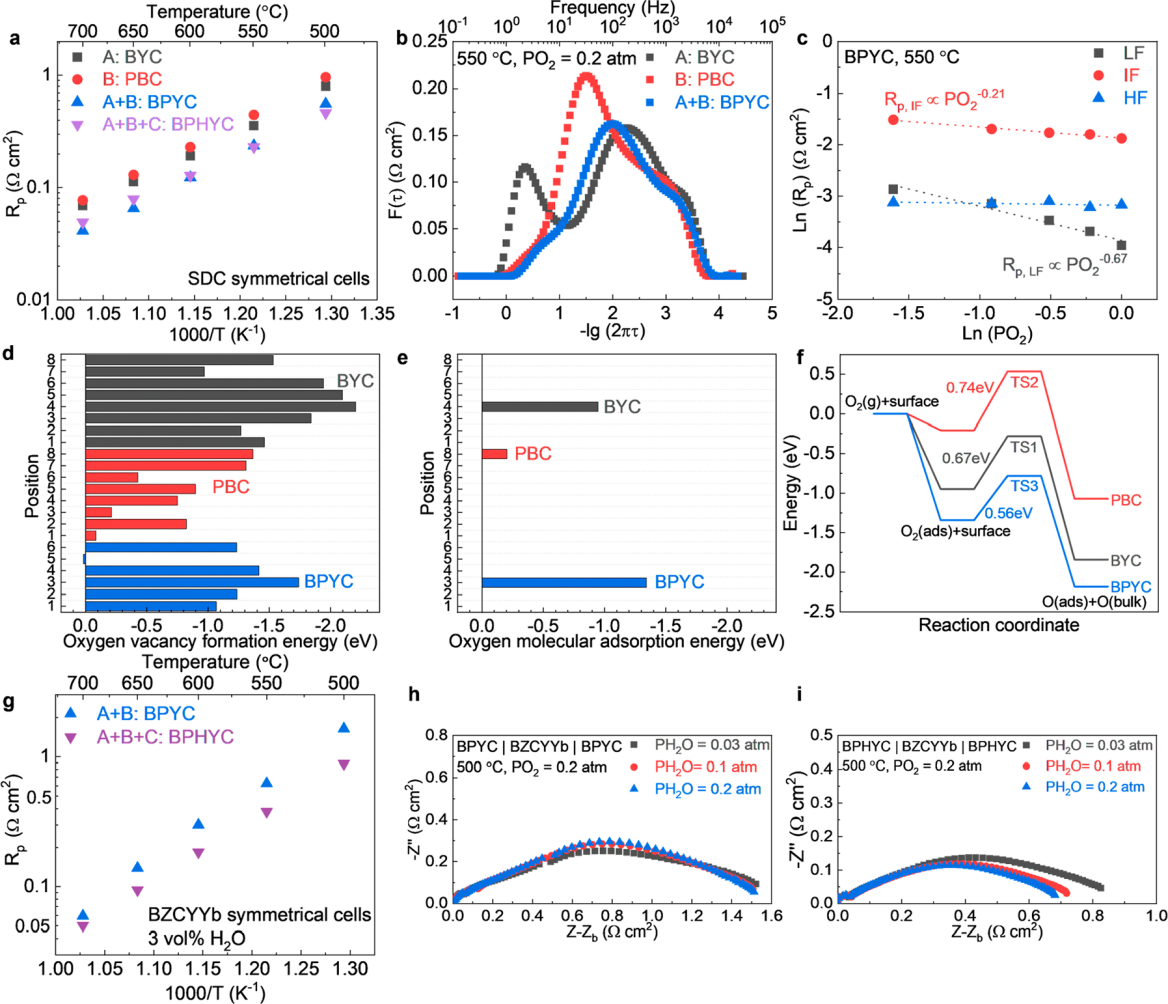
Synergistic effect of the three phases in BPHYC. (a) *R*_p_ values of BYC, PBC, BPYC, and BPHYC on SDC-based symmetrical cells. (b) DRT plots of BYC, PBC, and BPYC at 550 °C and an oxygen partial pressure of 0.2 atm. (c) Dependence of *R*_p_ at each frequency of BPYC as a function of *P*_O_2__ at 550 °C. (d) Oxygen vacancy formation energy at all possible sites on the BYC, PBC, and BPYC surfaces. (e) Oxygen molecular adsorption energy on the surfaces of BYC, PBC, and BPYC. (f) Energy change during the oxygen adsorption and dissociation processes on the surfaces of BYC, PBC, and BPYC. (g) *R*_p_ of BPYC and BPHYC on BZCYYb-based symmetrical cells with 3 vol % H_2_O. (h) EIS plot of the BPYC|BZCYYb|BPYC symmetrical cell at different concentrations of water at 500 °C (*P*_O_2__ = 0.2 atm). (i) EIS plot of the BPHYC|BZCYYb|BPHYC symmetrical cell at different concentrations of water at 500 °C (*P*_O_2__ = 0.2 atm).

To study the effect of BHY (phase C) in BPHYC on the ORR activity, the electrochemical performance of BPYC (A+B) and BPHYC (A+B+C) was compared on BZCYYb-based symmetrical cells. Figure 3g compares the *R*_p_ values of BPYC and BPHYC in wet air (with 3 vol % H_2_O), which shows the *R*_p_ value was reduced by adding BHY to BPYC. Considering that adding BHY to BPYC did not have a significant effect on the *R*_p_ values of SDC-based symmetrical cells (Figure 3a), the reduction of *R*_p_ by adding BHY to BPYC on BZCYYb-based symmetrical cells was most likely due to the improvement of proton-related ORR activity. Because water is the only source of protons in such a symmetrical cell configuration, we changed the water partial pressure while maintaining the oxygen partial pressure and compared the impedance of BPYC to BPHYC. As shown in Figure 3h and Figure S15a, with increasing water partial pressure from 0.03 to 0.2 atm, the *R*_p_ value of BPYC did not change at either 500 or 550 °C, indicating that higher concentrations of water do not accelerate the ORR kinetics of the BPYC electrode on a proton-conducting electrolyte. On the other hand, the *R*_p_ value of BPHYC continuously decreased as the water partial pressure was increased at both 500 and 550 °C (Figure 3i and Figure S15b). Because BZCYYb is a mixed conductor that supports transport of both proton and oxygen-ions, the reduction of *R*_p_ can originate either from the accelerated oxygen-ion-related ORR process or from the proton-related ORR process. As an example, in a previous study,^24^ Kim et al. reported that water can help generate a nanosized catalyst on the surface of certain electrode materials and improve the ORR kinetics. Because such a phenomenon was observed on an oxygen-ion-conducting symmetrical cell (using SDC as the electrolyte), proton did not contribute to the enhanced activity. To examine this possibility, we fabricated SDC-based BPHYC symmetrical cells. Because SDC is a pure oxygen-ion conductor, the contribution of proton conduction can be eliminated from the electrochemical process in SDC-based symmetrical cells. As shown in Figure S16, increasing the water partial pressure while maintaining the same oxygen partial pressure (*P*_O_2__ = 0.2 atm) did not affect the *R*_p_ value of BPHYC at 550 and 500 °C. These results indicate that increasing the water concentration does not cause an obvious enhancement of the ORR kinetics of the BPHYC electrode with an oxygen-ion-conducting electrolyte. Thus, the reduced *R*_p_ value of BPHYC at higher water partial pressures in Figure 3i and Figure S15b is due to proton-related ORR kinetics. Without the BHY phase, the performance of BPYC is insensitive to water partial pressure, indicating its limited activity. The water absorption capability of BHY was further investigated by theoretical calculations. Figure S17 shows the slab model of BHY with water adsorbed at both Hf and Y sites. The water adsorption energy on BHY was slightly lower than that on BZCYYb (Tables S4 and S5). Optimization of the ratio between these three phases was also conducted, as shown in Figures S18 and S19. The resulting optimized ratio was close to that of the BPHYC nanocomposite.

The synergistic effect of the BYC, PBC, and BHY phases on the excellent catalytic activity of BPHYC has been well-studied. For the next step, the triple-conducting property (electron, oxygen-ion, and proton) of BPHYC was evaluated to determine the intrinsic properties of this material. The total electrical conductivity of BPHYC as a function of temperature is shown in Figure 4a. Because the electronic conductivity is several orders of magnitude higher than the oxygen-ion conductivity and protonic conductivity, the total electrical conductivity can be approximately treated as the electronic conductivity. At 500 °C, the electronic conductivity of BPHYC is about 9 S cm^–1^, which is several times higher than those for other triple-conducting oxide materials (2 S cm^–1^ for BCCY and 1.2 S cm^–1^ for BCFZY).^6,13^ The oxygen transport properties of BPHYC were characterized by the electrical conductivity relaxation (ECR) measurement from 450 to 600 °C as shown in Figure 4b. At 500 °C, the surface kinetic coefficient (*k*_O_) and chemical diffusion coefficient of oxygen (*D*_O_) are 1.94 × 10^–4^ cm s^−1^ and 1.35 × 10^–5^ cm^2^ s^–1^, respectively. The proton transport properties of BPHYC were evaluated by the isotope exchange diffusion profile (IEDP).^25^ For a typical measurement, a dense BPHYC pellet was first annealed in 10% H_2_O for 24 h to reach equilibrium. Then the atmosphere was switched to 10% D_2_O, and the sample was treated for another 1 h. The proton concentration profile was measured by time-of-flight secondary ion mass spectrometry (ToF-SIMS).^25,26^Figure S20 shows the OD concentration profile in the BPHYC sample. The surface kinetic coefficient (*k*_H_) and self-diffusion coefficient (*D*_H_) are shown as functions of temperature in Figure 4c. As the temperature increases, *D*_H_ and *D*_O_ both increase due to faster kinetics. However, *k*_H_ decreases as the temperature increases. This is due to the exothermic properties of proton uptake at the surface, which have been reported for both proton-conducting electrolyte materials and other triple-conducting air electrode materials.^14,25,26^ The long-term stability of BPHYC was further evaluated in symmetrical cells. As shown in Figure 4d,e, BPHYC demonstrated good electrochemical stability in humidified air on both SDC and BZCYYb electrolytes at 500 and 550 °C for up to 500 h. The stability of BPHYC under 30 vol % H_2_O was also confirmed on BZCYYb-based symmetrical cells (Figure S21). The triple-conducting properties and stability further confirm the potential of BPHYC for use as an air electrode material in R-PSOCs.

**Figure 4:**
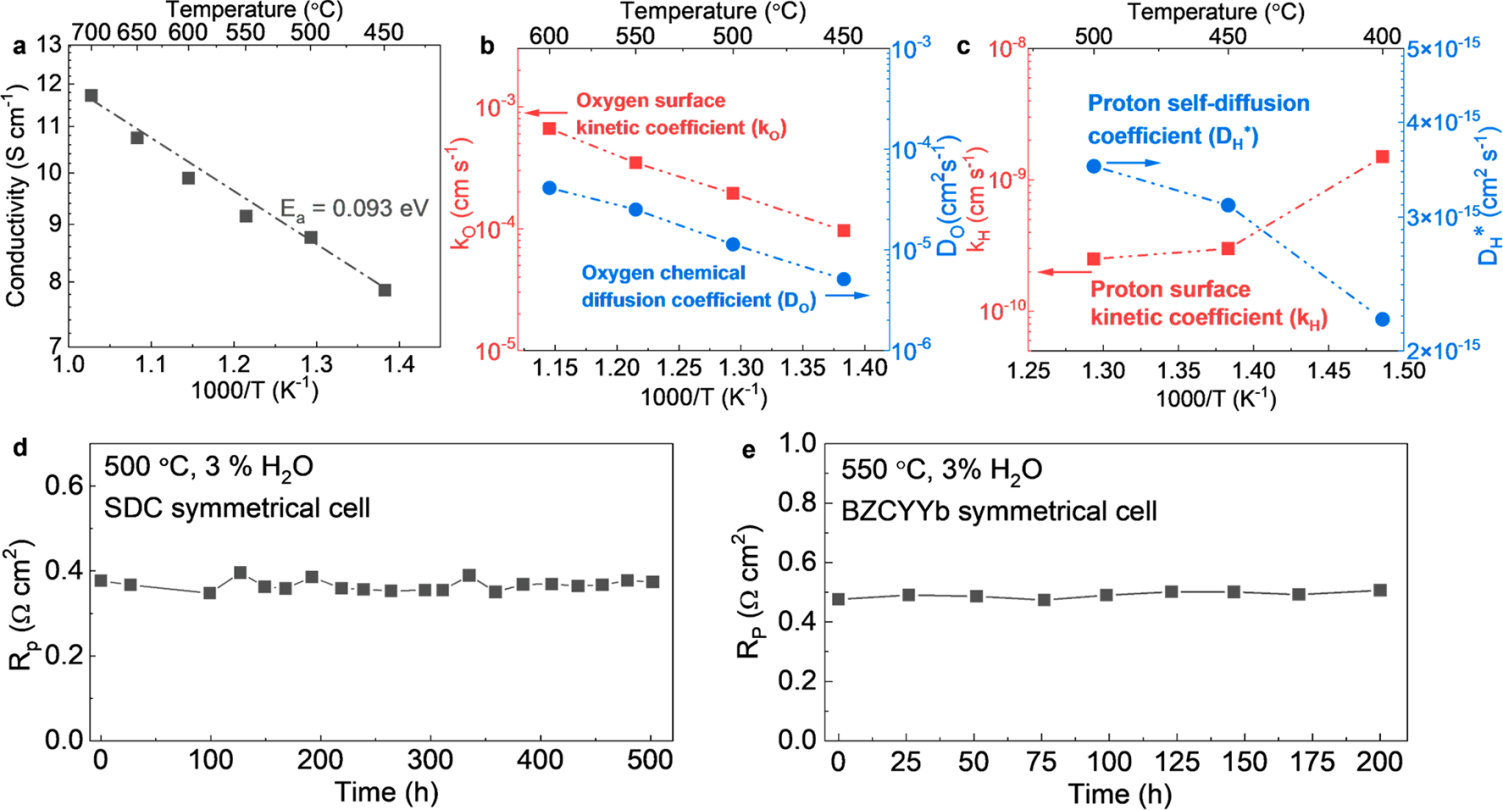
Conductivity and stability of BPHYC. (a) Total electrical conductivity of BPHYC. (b) Oxygen surface kinetic coefficient and chemical diffusion coefficient measured by ECR. (c) Proton surface kinetic coefficient and self-diffusion coefficient measured by IEDP. (d) *R*_p_ of BPHYC on SDC-based symmetrical cells in humidified air (3 vol % H_2_O) at 500 °C for 500 h. (e) *R*_p_ of BPHYC on BZCYYb-based symmetrical cells in humidified air (3 vol % H_2_O) at 550 °C for 200 h.

Single cells with BPHYC air electrodes were fabricated to evaluate the material’s electrochemical performance and durability in fuel cell mode, electrolysis mode, and reversible mode. The cross-sectional view of a single cell with a configuration of Ni-BZCYYb|BZCYYb|BPHYC is shown in Figure 5a. The thickness of the electrolyte was approximately 10 μm. A robust air electrode and electrolyte interface (without delamination) was shown, likely due to minimal mismatch in the thermal expansion coefficient of BPHYC with that of the electrolyte. EIS plots of the single cell under the OCV condition are shown in Figure 5b. At 500 °C, the *R*_p_ value of the single cell was only 0.518 Ω cm^2^, smaller than those of most of the other cells reported to date.^3,12,14^ As shown in Figure 5c, when operated in fuel cell mode, using hydrogen as the fuel and air as the oxidant, the peak power densities were 1.37, 0.96, and 0.62 W cm^–2^ at 600, 550, and 500 °C, respectively. This performance is better than those of most other state-of-the-art air electrode materials in the fuel cell mode with a similar cell configuration reported to date (same electrolyte material with a similar thickness, without any other modifications on the electrodes or interfaces), as compared in Figure 5d. In the electrolysis mode, an electrolysis current density of 2.40 A cm^–2^ was achieved at an applied voltage of 1.3 V at 600 °C, surpassing that of other state-of-the-art air electrode materials (Figure 5e ,f).

**Figure 5:**
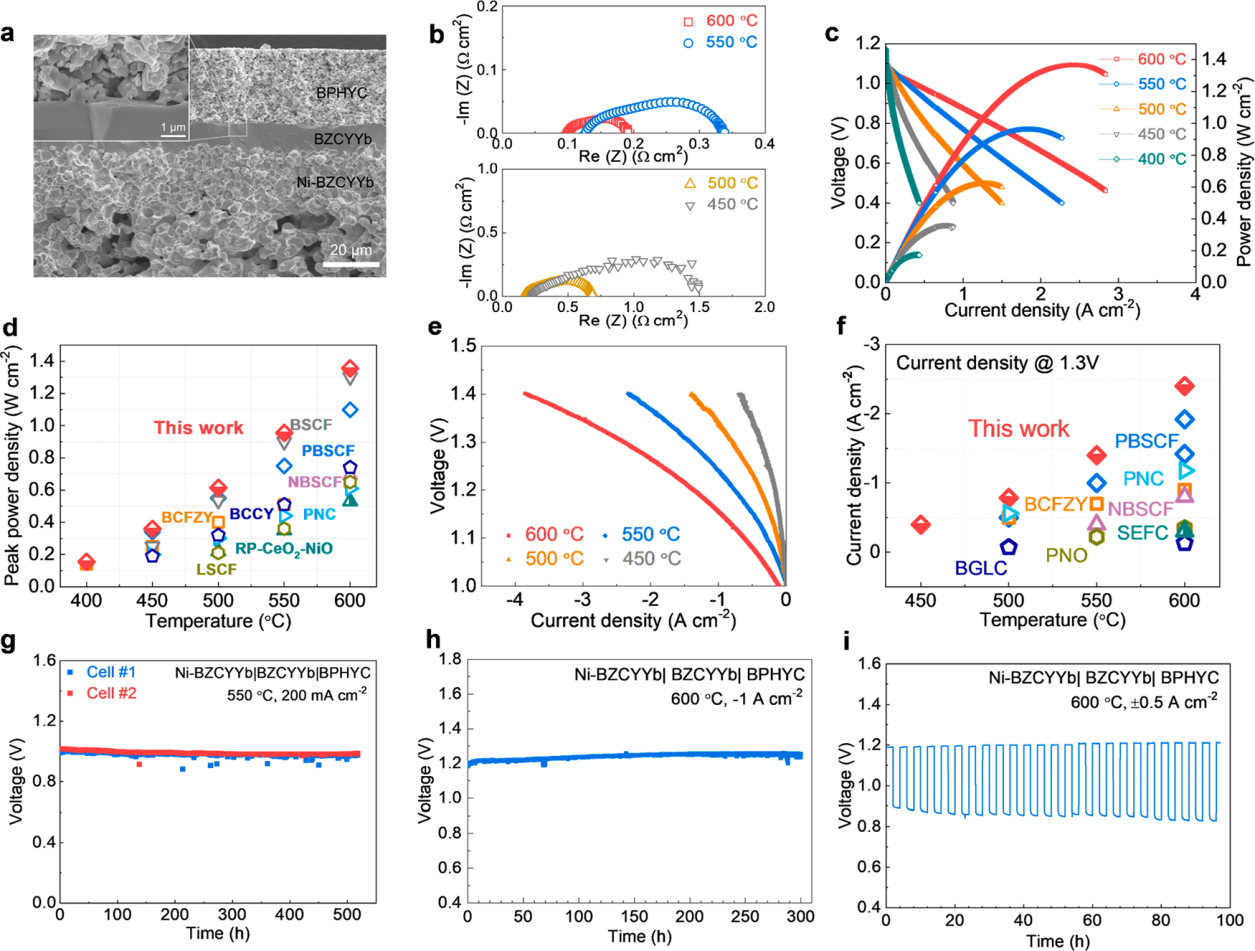
Electrochemical performance and stability of BPHYC applied to BZCYYb-based R-PSOCs. (a) Cross-sectional SEM image of the Ni-BZCYYb|BZCYYb|BPHYC single cell before testing. Inset: high-magnification SEM image of the interface between the air electrode and the electrolyte. (b) EIS plots of the single cell at OCV. (c) *I*–*V*–*P* curves in the fuel cell mode using hydrogen as the fuel and air as the oxidant at different temperatures. Fuel electrode atmosphere: 20 sccm 3% H_2_O–H_2_. Air electrode atmosphere: 100 sccm air. (d) Power density comparison of BPHYC with other state-of-the-art air electrode materials under similar testing conditions: BSCF,^27^ PBSCF (PrBa_0.5_Sr_0.5_Co_1.5_Fe_0.5_O_5+δ_),^28^ PNC (PrNi_0.5_Co_0.5_O_3−δ_),^29^ NBSCF (NdBa_0.5_Sr_0.5_Co_1.5_Fe_0.5_O_5+δ_),^30^ BCFZY,^31^ and BCCY.^13^ (e) *I*–*V* curves of the electrolysis cell with BPHYC as the air electrode at different temperatures. Fuel electrode atmosphere: 20 sccm 3% H_2_O–H_2_. Air electrode atmosphere: 100 sccm 30% H_2_O–air. (f) Electrolysis current density comparison of BPHYC with other state-of-the-art air electrode materials at an applied voltage of 1.3 V: SEFC (SrEu_2_Fe_1.8_Co_0.2_O_7−δ_),^32^ BGLC (BaGd_0.8_La_0.2_Co_2_O_5+δ_),^8^ PBSCF,^28^ PNC,^29^ NBSCF,^30^ BCFZY,^31^ and PNO (Pr_2_NiO_4+δ_).^33^ (g) Stability of two single cells operated in the fuel cell mode at 550 °C for over 500 h. Fuel electrode atmosphere: 20 sccm 3% H_2_O–H_2_. Air electrode atmosphere: ambient air. (h) Stability of a single cell operated in the electrolysis mode at 600 °C. Fuel electrode atmosphere: 20 sccm 3% H_2_O–H_2_. Air electrode atmosphere: 100 sccm 30% H_2_O–air. (i) Stability of a single cell operated in the reversible mode at 600 °C. Fuel electrode atmosphere: 20 sccm 3% H_2_O–H_2_. Air electrode atmosphere: 100 sccm 3% H_2_O–air.

The stability of BPHYC was evaluated on single cells under actual operating conditions. As shown in Figure 5g, two cells were operated at 550 °C in the fuel cell mode to examine the electrochemical stability. Both cells operated stably for over 500 h, confirming the durability and repeatability. As shown in Figure S22, a cell was operated at 500 °C for over 200 h, which demonstrates relatively worse stability at a lower temperature that is most likely due to the more fragile interface between the electrolyte and the air electrode.^34^ These stability results are among the best reported to date at temperatures below 600 °C. The durability in the electrolysis mode was confirmed under 30 vol % H_2_O at 600 °C with an electrolysis current density of 1 A cm^–2^ for 300 h, as shown in Figure 5h. The microstructure of the single cell was preserved after the long-term stability test under electrolysis mode (Figure S23). As shown in Figure 5i, the single cell operated reversibly in both the fuel cell mode to generate electricity and the electrolysis mode to produce hydrogen for 100 h at 600 °C. These electrochemical results further confirmed BPHYC as a promising air electrode material for R-PSOCs at intermediate temperatures.

In this study, a series of air electrode materials have been designed by introducing transition- and rare-earth-metal ions into the BHY electrolyte material. When used as the electrode in BZCYYb-based symmetrical cells, the BPHYC electrode shows the best electrocatalytic activity and the lowest polarization resistance (*R*_p_) ever reported when compared with other air electrode materials. A detailed analysis revealed that BPHYC consists of three different phases, BYC, PBC, and BHY. Impedance analysis (DRT) indicated that the BYC phase contributed to the bulk oxygen-ion conduction, the PBC phase facilitated the surface oxygen adsorption and dissociation, and the BHY phase accelerated the kinetics of electrode reactions involving protons. These three phases play a synergistic role in enhancing the electrochemical performance of the BPHYC electrode. Additionally, the oxygen and proton transport kinetics were evaluated using ECR and IEDP. Single cells with the BPHYC air electrode demonstrated outstanding performance in both the fuel cell and electrolysis modes. Finally, the long-term stability of BPHYC was confirmed on both symmetrical cells and single cells under various operating conditions for over 500 h.

## Supporting Information Available

Experimental section, XRD, DFT calculations, electrochemical analysis, and other long-term durability results (PDF)
