# Supplementary material for: A Synergistic Three-Phase, Triple-Conducting Air Electrode for Reversible Proton-Conducting Solid Oxide Cells
Source: ACS Energy Lett. 2023 Sep 1;8(10):3999–4007. doi: 10.1021/acsenergylett.3c01251 (PMC10580316; doi:10.1021/acsenergylett.3c01251)
Supplement: Supplementary file 1 — nz3c01251_si_001.pdf [file nz3c01251_si_001.pdf]

Supporting Information

A Synergistic Three-Phase, Triple-Conducting Air Electrode for Reversible Proton-Conducting Solid Oxide Cells

Weilin Zhang,[1] Yucun Zhou,[1] Xueyu Hu,[1] Yong Ding,[1] Jun Gao,[2] Zheyu Luo,[1] Tongtong Li,[1,3] Nicholas Kane,[1] Xiao-Ying Yu,[4] Tanguy Terlier,[5] and Meilin Liu[1]**

[1] School of Materials Science and Engineering, Georgia Institute of Technology, Atlanta, GA, 30332-0245, USA.

[2] Energy and Environment Directorate, Pacific Northwest National Laboratory, Richland, WA, 99354, USA

[3] Energy Materials and Surface Sciences Unit, Okinawa Institute of Science and Technology Graduate University, 1919-1 Tancha, Onna-son, Kunigami-gun, Okinawa 904-0495, Japan

[4] Materials Science and Technology Division, Oak Ridge National Laboratory, Oak Ridge, TN, 99354, USA

[5] Shared Equipment Authority, SIMS Laboratory, Rice University, Houston, TX, 77005, USA.

Corresponding Author

* Meilin Liu: meilin.liu@mse.gatech.edu, Yucun Zhou: zhouycwf@gmail.com

Experimental Section/Methods

Synthesis of air electrode powder

Air electrode powder was synthesized by the solid-state reaction (SSR) method (Figure S1). For example, to synthesize BPHYC, stoichiometric amounts of $BaCO_3$, Pr_6O_{11}, HfO_2, Y_2O_3, and Co_3O_4 were ball milled in ethanol for 12 hours. The powder was then dried, pressed, and fired at 1050 °C for 12 hours to achieve the desired phase. The powder was further ball milled for 24 hours for further ink preparation. $PrBaCo_2O_{5+\delta}$ (PBC), Y doped $BaCoO_{3-\delta}$ (BYC), and Y doped $BaHfO_{3-\delta}$ (BHY) were synthesized and prepared in the same method. The phase of each powder was characterized by X-ray diffraction (XRD).

Fabrication of symmetrical cells and single cells

To fabricate symmetrical cells, SDC and BZCYYb powder were mixed with 1 wt% PVB and then dry pressed and sintered at 1450 °C for 5 hours (1 wt% of NiO was also added to BZCYYb powder for symmetrical cell fabrication). Air electrode ink (air electrode powder mixed with V-006) was brush painted on both sides of the electrolyte followed by firing at 950 °C for 2 hours. To fabricate single cells, Ni-BZCYYb half cells were prepared by tape casting and sintering at 1450 °C for 5 hours, which is described elsewhere.[1, 2] The air electrode ink was brush painted onto the BZCYYb electrolyte and fired at 950 °C for 2 hours. The effective area of the symmetrical cells and single cells was 0.28 cm^2.

Electrochemical measurements

For symmetrical cell measurements, two pieces of silver mesh were used as the current collectors. Impedance spectra were acquired using a Solartron 1255 HF frequency response analyzer interfaced with an EG&G PAR potentiostat model 273A with an AC amplitude of 10 mV in the frequency range from 100 kHz to 0.01 Hz. The stability testing of the symmetrical cells was performed at 550 and 500 °C under the open circuit voltage (OCV) with different concentrations of H_2O on both SDC and BZCYYb electrolytes. For the single cell measurement, 20 sccm wet hydrogen (with 3 vol% H_2O) was used as the fuel and 100

sccm air was used as the oxidant. For the electrolysis cell measurement, 20 sccm wet hydrogen (with 3 vol% H_2O) was used in the fuel electrode and 100 sccm wet air (with different concentrations of water) was used in the air electrode. The cell performance and stability were monitored with an Arbin multi-channel electrochemical testing system.

Other characterizations

The phase structure of the air electrode powder was characterized by X-ray diffraction (Panalytical XPert PRO Alpha-1 XRD). The microstructure and morphology of the cells were examined by a scanning electron microscope (SEM, Hitachi SU8010). The crystal structure of the different phases of the air electrode was characterized with a scanning transmission electron microscope (STEM, Hitachi HD-2700). The oxygen surface kinetic coefficient (k_O) and chemical diffusion coefficient (D_O) were characterized by the electrical conductivity relaxation (ECR) measurement. Proton surface kinetic coefficient (k_H) and self-diffusion coefficient (D_H) were evaluated by the isotope exchange diffusion profile (IEDP) method.[3] BPHYC was first pressed into a pellet and densified by sintering at 1225 °C for 5 hours. For a typical measurement, the dense BPHYC pellet was first annealed in 10% H_2O for 24 hours to achieve equilibrium. Then the atmosphere was switched to 10% D_2O and treated for another 1 hour. The proton concentration profile was measured by time-of-flight secondary ion mass spectrometry (ToF-SIMS).

Computational details

All spin-polarized calculations were performed with density functional theory (DFT) method using the Vienna ab initio simulation package (VASP).[4, 5] The projector augment wave (PAW) method was applied with Hf([Kr]$5p^6 5d^2 6s^2$), Pr([Kr]$5s^2 5p^6 6s^2$) Ba([Kr]$5s^2 5p^6 6s^2$), Y([Ar]$4s^2 4p^6 4d^1 5s^2$), Co([Ne]$3d^7 4s^2$), and O([He]$2s^2 2p^4$) to solve the interaction between ionic core electrons and valence electrons. The generalized gradient approximation (GGA) with Perdew-Butke-Ernzerhof (PBE) functional was used to take the exchange-correlations into consideration in the Kohn-Sham equations.[6] GGA+U with U_{eff} = 3.3 eV was used in the calculations to describe the correlated electrons of the Co 3d-orbital.[7] The energy cutoff and

convergence criteria were set as 520 eV and 10^{-5} eV, respectively. The structures were relaxed until the force on each atom less than 0.02 eV $Å^{-1}$. The RMM-DIIS algorithm and the conjugate-gradient were used during the electronic and ionic optimization, respectively. For the pristine PBC, a supercell of PBC with a size of $2a \times 2a \times 2a$ was constructed, containing 4 Pr, 4 Ba, 8 Co, and 24 O atoms, to describe its properties. For the pristine BYC, a supercell of $BaCo_{0.875}Y_{0.125}O_3$ with a size of $2a \times 2a \times 2a$ was constructed, containing 8 Ba, 7 Co, 1 Y, and 24 O atoms, to approximately describe its properties. A $3 \times 3 \times 3$ Γ-centered k-point sampling grid was chosen for Brillouin zone integration. To elucidate the activity of PBC and BYC for oxygen reduction reaction (ORR), the most active low-index (001) surface was cleaved with BO_2 surface terminated.[8] Hence, an eight-layer $2a \times 2a$ PBC (001) and BYC (001) BO_2-terminated slab, with the bottom four layers fixed, was built as the substrate for catalysis investigation. And a vacuum layer of 15 Å was set to avoid the inter-slab interaction between two neighboring cells. Dipole correction was applied and a $3 \times 3 \times 1$ Γ-centered k-point sampling grid was chosen for the Brillouin zone integration. To further shed light on the BYC-PBC heterostructure, the bulk BYC and PBC were merged together with a ratio of 1:1, to build the BYC-PBC interface. Similarly, the most active low-index (001) surface was cleaved for ORR investigation.

Detailed pathways of the ORR and energy of the transition state (TS) were simulated with the climbing image nudged elastic band (CI-NEB) method.[9] In this case, four to six intermediate images were used with the forces minimized to 0.03 eV $Å^{-1}$. Microkinetic analysis based on the transition state theory was conducted to validate the calculation.[10] We systematically investigated all the possible rate-determinant elementary steps for ORR, and the most favorable pathways were discussed in detail. Calculations of the O_2 molecules have been performed in advance. Bader charges were also calculated to illustrate the charge transfer information. The oxygen vacancy formation energy is defined as

$$E_v = E(defect\ slab) + 1/2E(O_2) - E(perfect\ slab) \qquad (1)$$

where $E(defect\ slab)$ is the total energy of the defect surface with one oxygen vacancy, $E(O_2)$ is the total energy of free oxygen molecular, and $E(perfect\ slab)$ is the total energy of perfect surface. The oxygen adsorption energy is defined as

$$E_{ads-O_2} = E(slab - O) - E(O_2) - E(defect\ slab) \qquad (2)$$

where $E(slab - O)$ is the total energy of the perfect surface with one extra oxygen atom adsorbed on the surface cobalt atom.

For the pristine $BaHf_{0.5}Y_{0.5}O_{3-\delta}$, a supercell of $BaHf_{0.5}Y_{0.5}O_3$ with a size of $2\sqrt{2}a \times 2\sqrt{2}a \times 2a$ was constructed, containing 16 Ba, 8 Hf, 8 Y and 48 O atoms, to describe its properties. A $2 \times 2 \times 3$ Γ-centered *k*-point sampling grid was chosen for Brillouin zone integration. To elucidate the surface property of $BaHf_{0.5}Y_{0.5}O_3$, an eight-layer $2\sqrt{2}a \times 2\sqrt{2}a$ $BaHf_{0.5}Y_{0.5}O_3$ (001) AO-terminated surface was built as the substrate for water adsorption. A vacuum layer of 15 Å was set to avoid the inter-slab interaction between two neighboring cells. Dipole correction was applied and a $3 \times 3 \times 1$ Γ-centered *k*-point sampling grid was chosen for the Brillouin zone integration. Calculations of the H_2O molecules have been performed in advance. The water adsorption energy is defined as

$$E_{ads-H_2O} = E(slab - 2H) - E(H_2O) - E(perfect\ slab) \qquad (3)$$

where $E(ads - 2H)$ is the total energy of the perfect surface with two extra hydrogen atoms adsorbed on oxygen atoms, $E(H_2O)$ is the total energy of free water molecular, and $E(perfect\ slab)$ is the total energy of perfect surface.For comparison, the hydration energy and water adsorption energy of BZCYYb1711 were considered.[11]

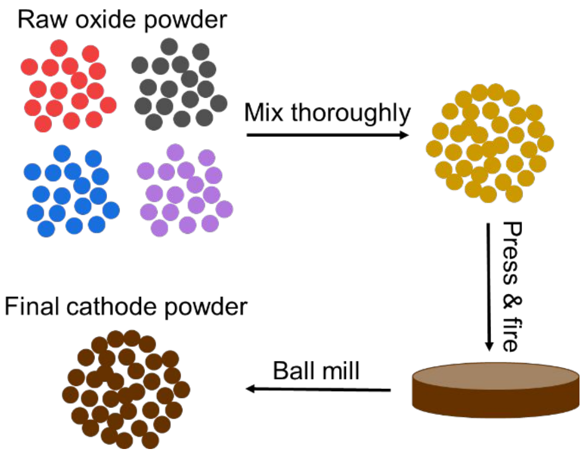

Figure S1. Synthesis of air electrode material candidates via the solid-state reaction method. Stoichiometric amounts of oxides and carbonate were mixed thoroughly in ethanol for 12 hours. The powder was then dried, pressed, and fired at 1050 °C for 12 hours to achieve the desired phase.

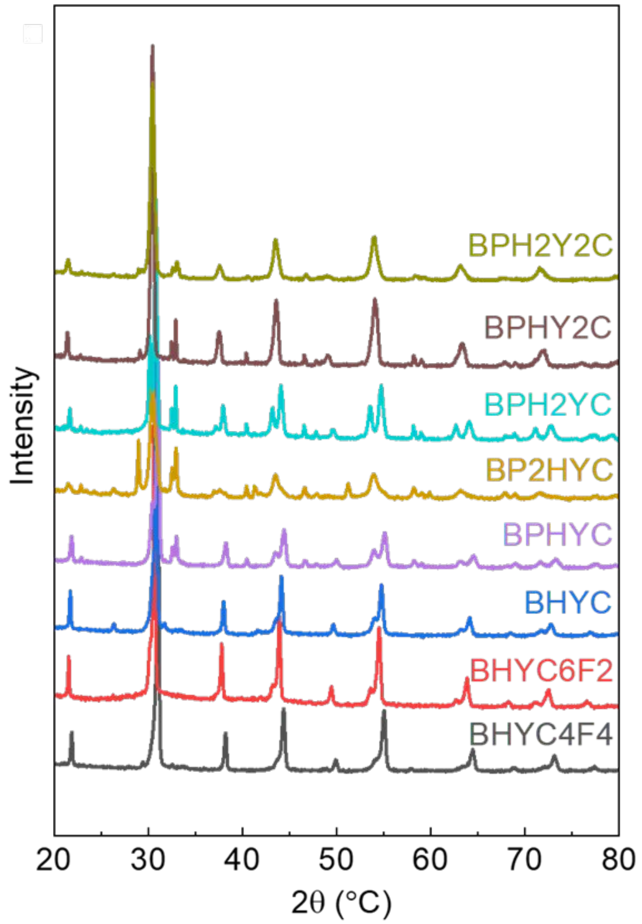

Figure S2. XRD patterns of candidate air electrode materials investigated. These materials were synthesized via the solid-state reaction method and fired in air at 1050 °C for 12 hours.

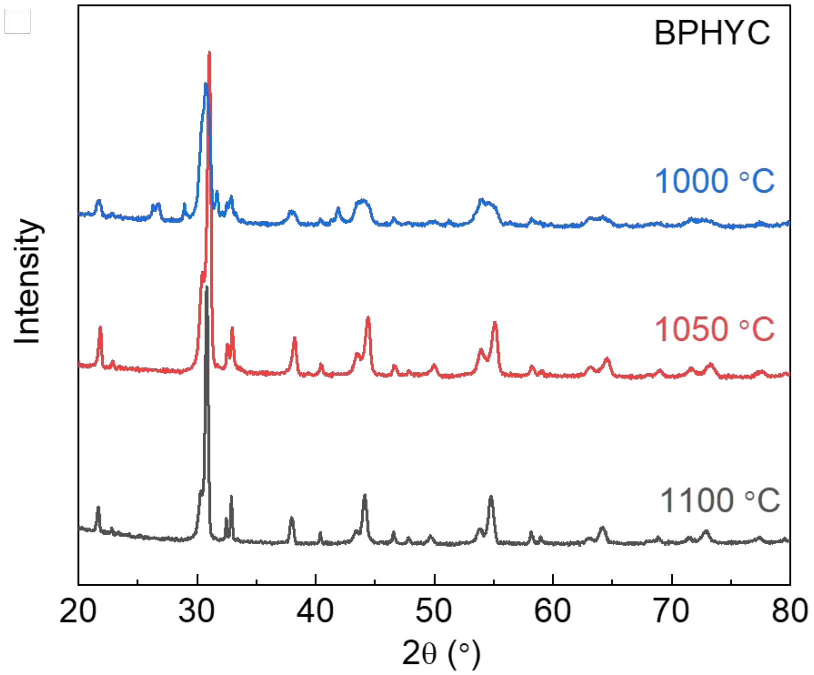

Figure S3. XRD patterns of BPHYC after firing at different temperatures for 12 hours. Firing at 1050 °C for 12 hours successfully achieves the three phases.

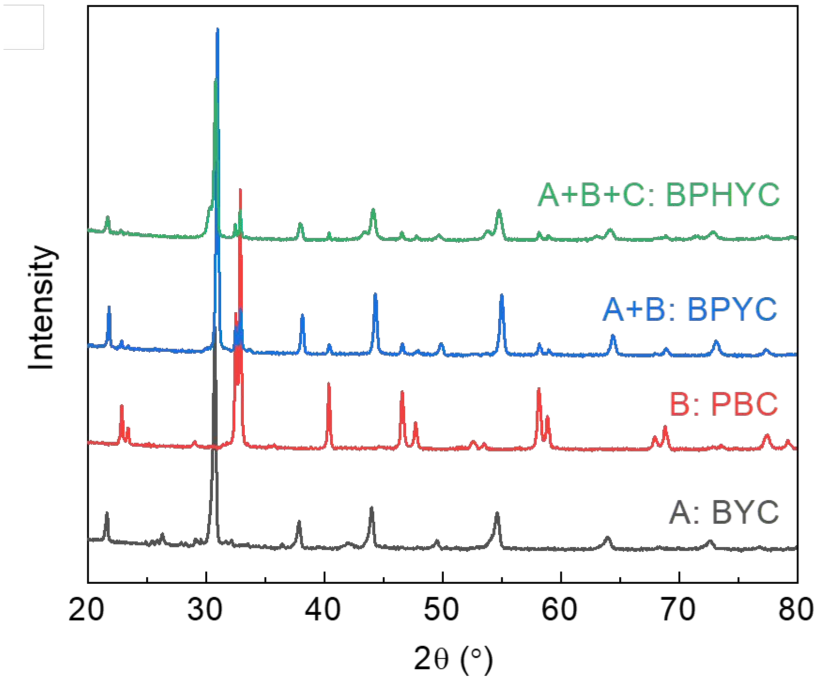

Figure S4. XRD patterns of single-phase BYC (phase A), PBC (phase B), the mixture of BYC and PBC (BPYC, A+B), and the mixture of BYC, PBC, and BHY (BPHYC, A+B+C).

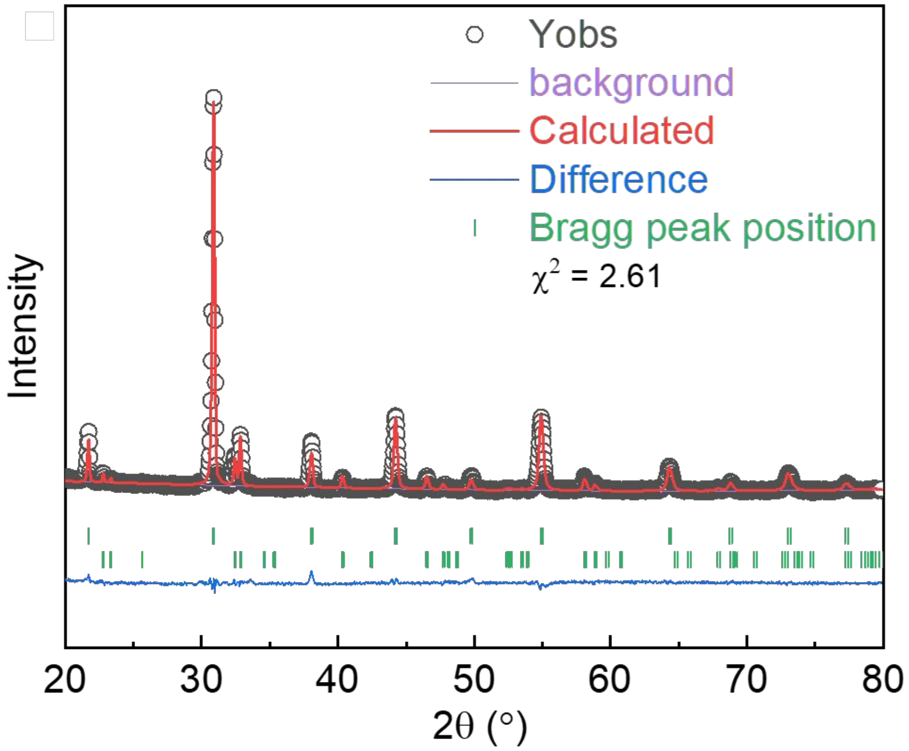

Figure S5. XRD refinement results of BPYC. BYC (phase A) and PBC (phase B) phases were identified. The mass ratio between BYC and PBC is similar to that in BPHYC (Table S2 and Table S3).

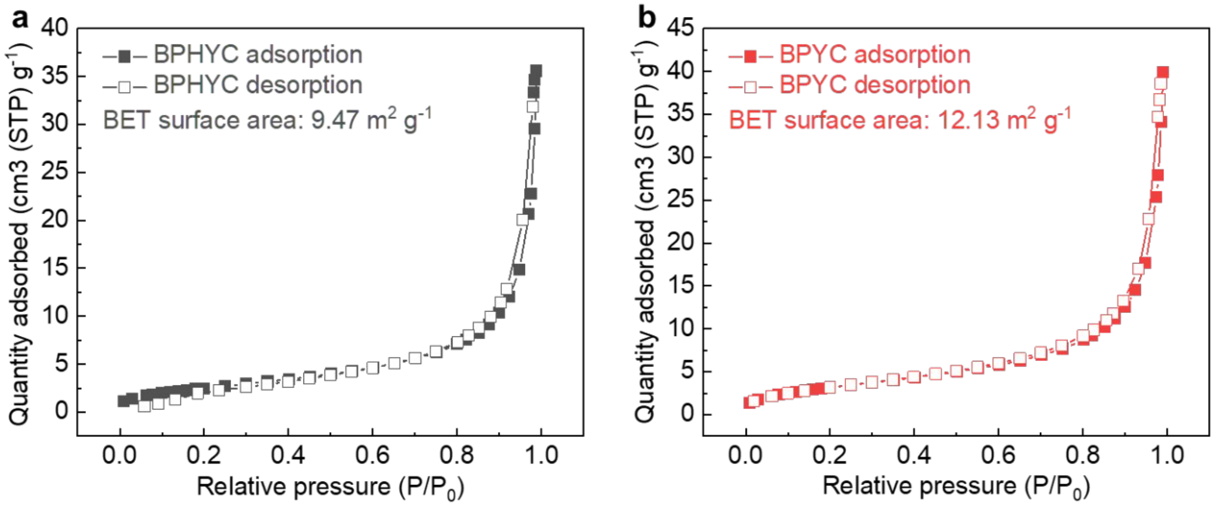

Figure S6. The nitrogen adsorption-desorption isotherm plots and Brunauer–Emmett–Teller (BET) surface area of (a) BPHYC and (b) BPYC powder. BPHYC and BPYC powder have a comparable surface area.

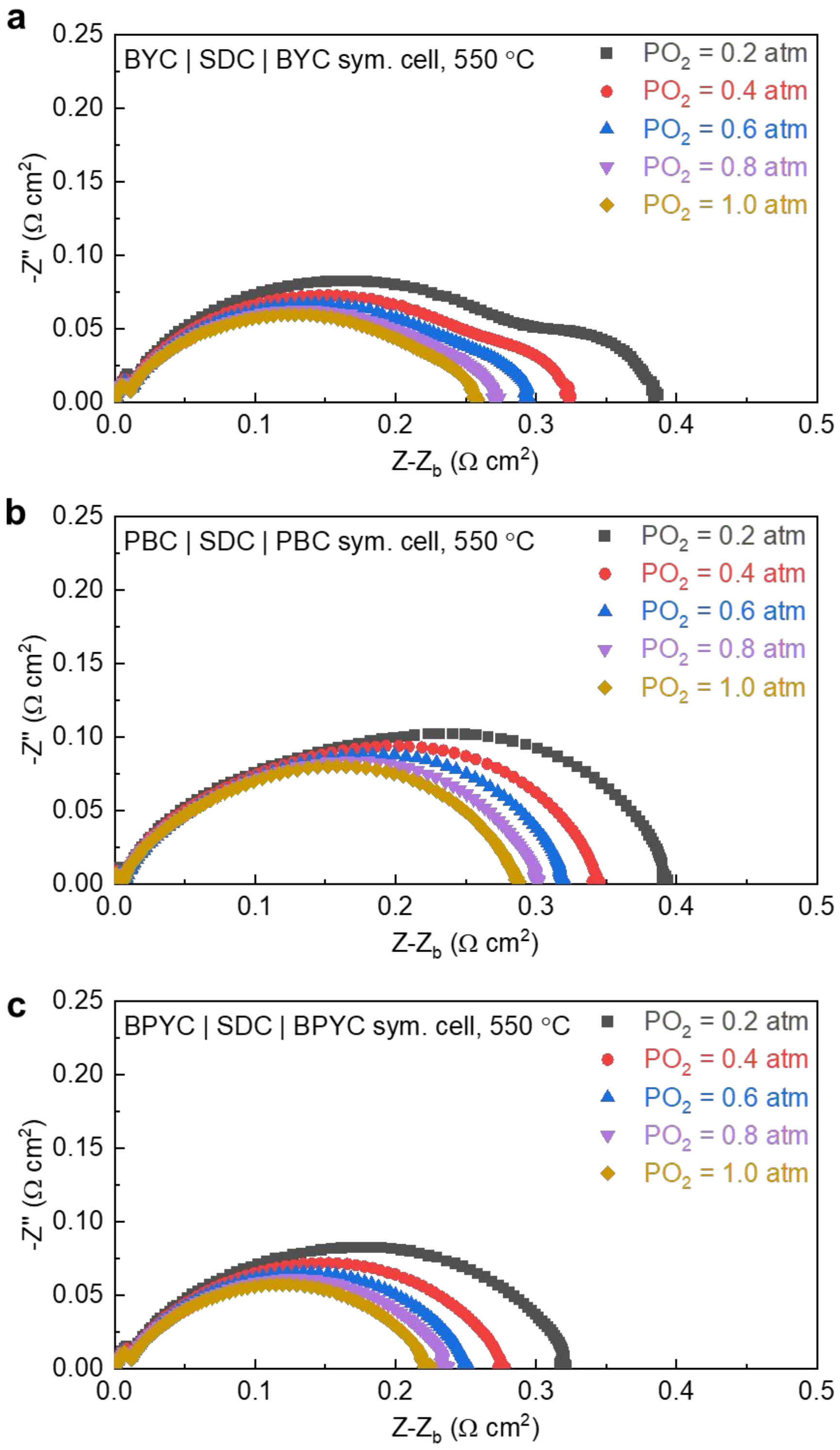

Figure S7. Electrochemical impedance spectroscopy (EIS) plots of BYC, PBC, and BPYC symmetrical cells as a function of oxygen partial pressure (P_{O2}) at 550 °C. R_p of all three electrodes decrease as P_{O2} increases from 0.2 atm to 1.0 atm.

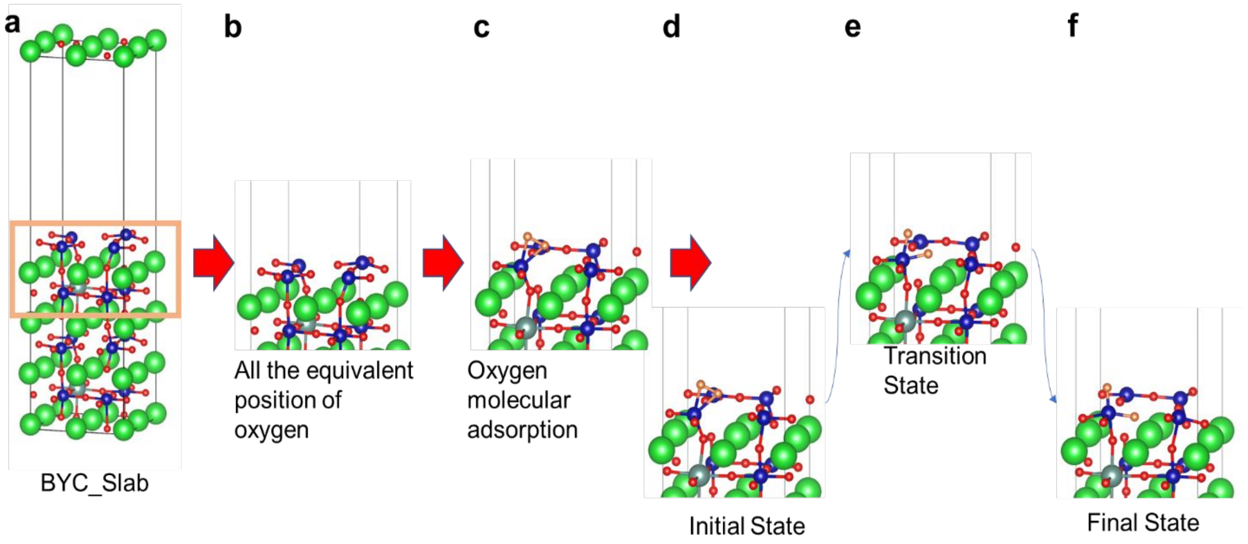

Figure S8. Simulation of ORR on BYC surface. (a) Slab model of BYC; (b) all oxygen sites on the BYC surface; (c) adsorption of oxygen molecule to BYC surface; (d-f) oxygen dissociation pathway on BYC surface, including initial state, transition state, and final state. The corresponding energy change is shown in Figure 3f.

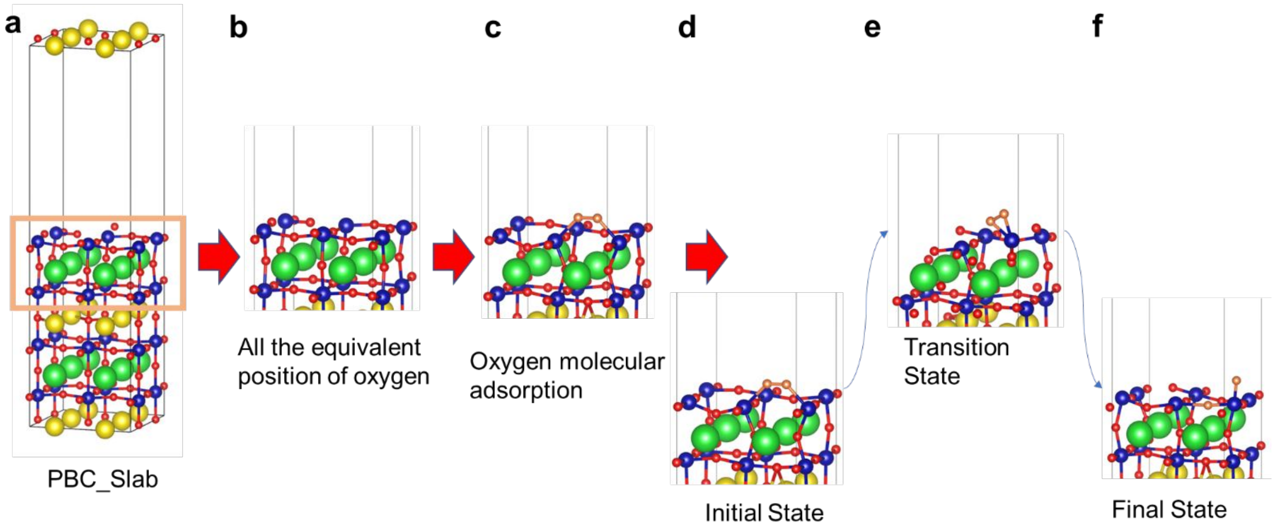

Figure S9. Simulation of ORR on PBC surface. (a) Slab model of PBC; (b) all oxygen sites on the PBC surface; (c) adsorption of oxygen molecule to PBC surface; (d-f) oxygen dissociation pathway on PBC surface, including initial state, transition state, and final state. The corresponding energy change is shown in Figure 3f.

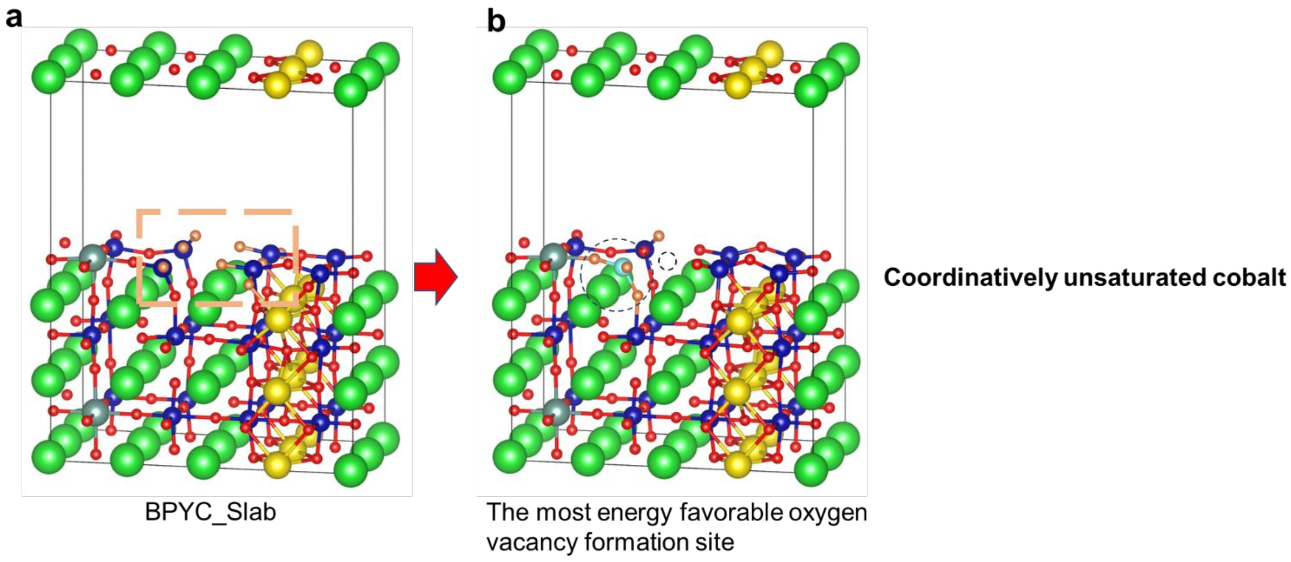

Figure S10. (a) Slab model of BPYC surface. (b) Coordinatively unsaturated cobalt site on BPYC surface after formation of oxygen vacancy, which is beneficial for the oxygen adsorption and further reaction.

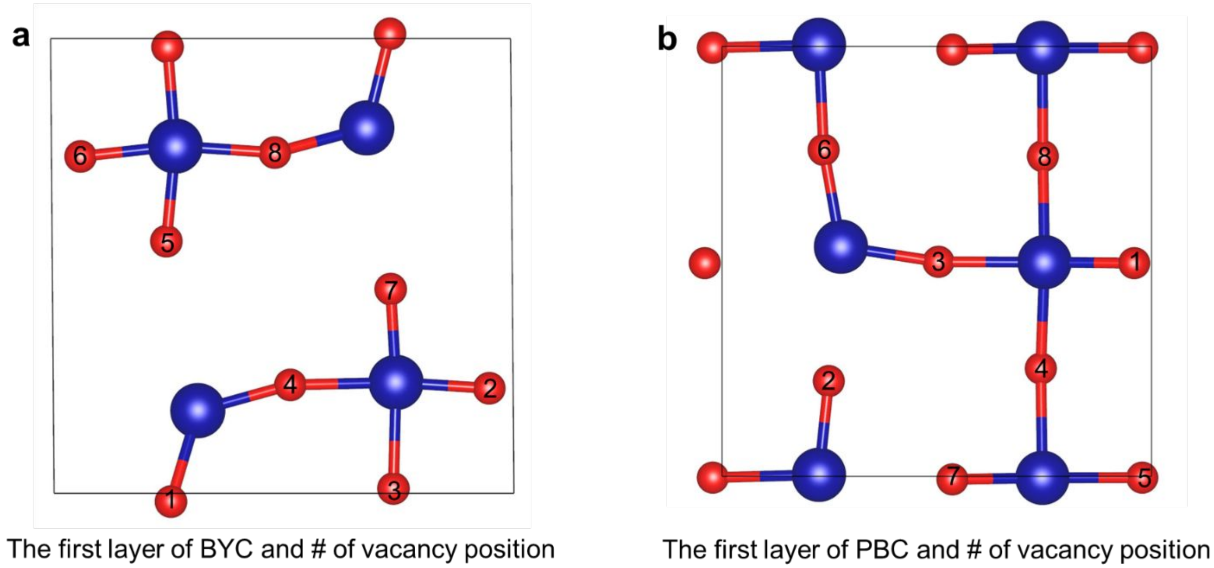

Figure S11. Top view of the BYC (a) and PBC (b) surface and all possible oxygen vacancy positions (corresponding to the number in Figure 3d and 3e).

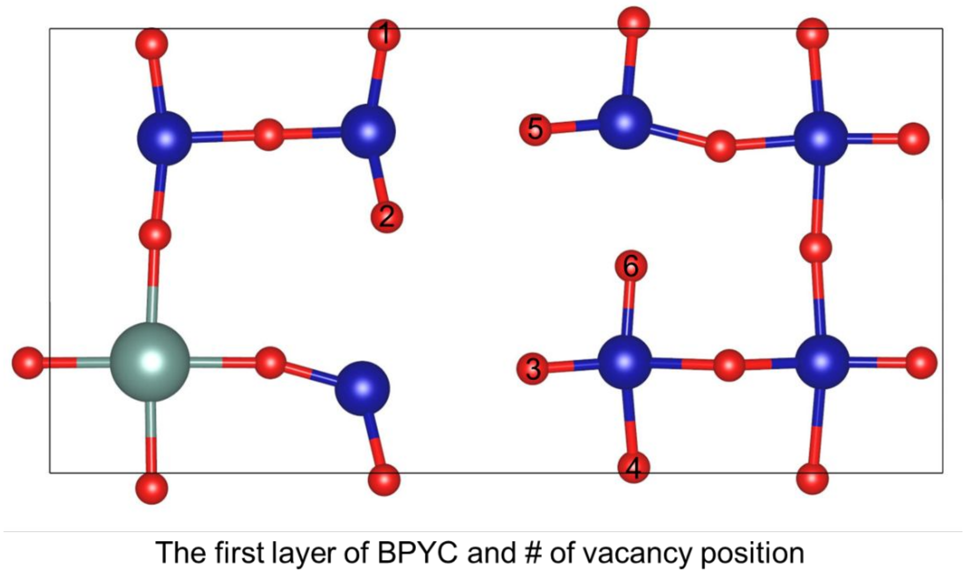

Figure S12. Top view of the BPYC surface and all possible oxygen vacancy positions (corresponding to the number in Figure 3d and 3e).

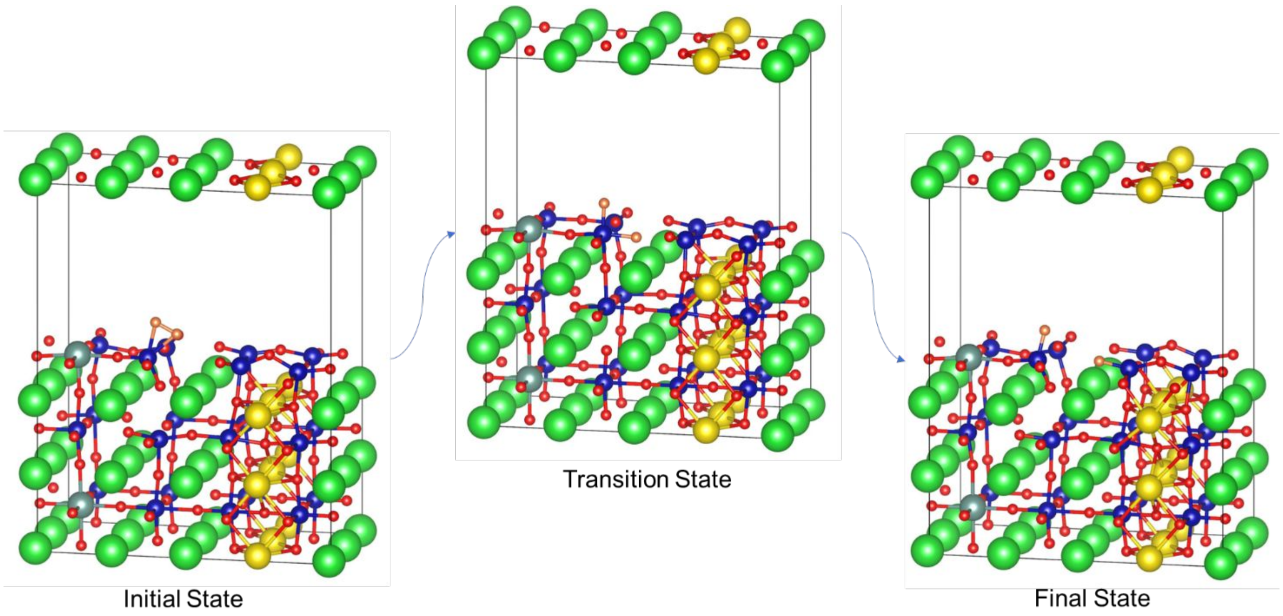

Figure S13. Oxygen dissociation pathway on BPYC surface, including initial state, transition state, and final state. The corresponding energy change is shown in Figure 3f.

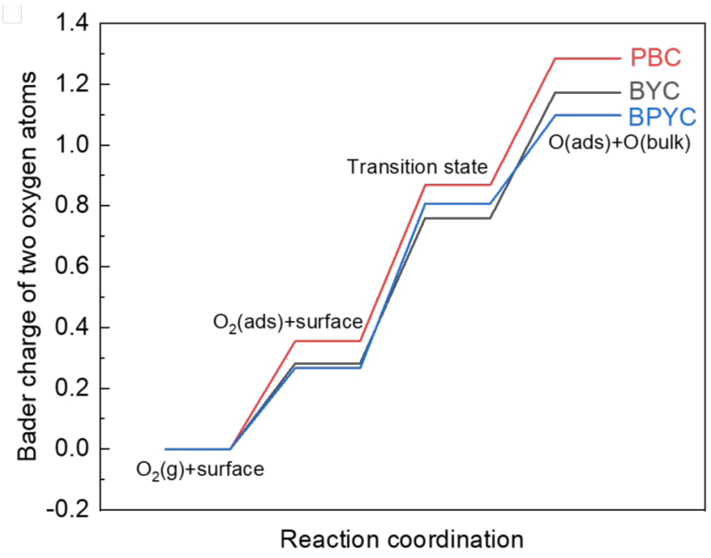

Figure S14. Bader charge during the oxygen dissociation reaction of BYC, PBC, and BPYC electrodes. The oxygen dissociation process in our theoretical calculation also includes the charge transfer process in the DRT analysis.

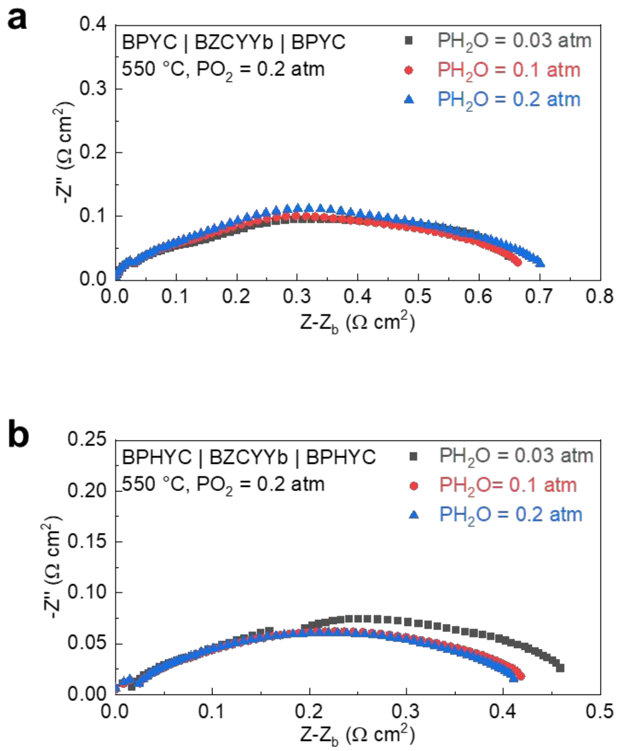

Figure S15. (a) EIS plots of the BPYC|BZCYYb|BPYC symmetrical cell at different concentrations of water at 550 °C (PO_2 = 0.2 atm). (b) EIS plots of the BPHYC|BZCYYb|BPHYC symmetrical cell at different concentrations of water at 550 °C (PO_2 = 0.2 atm).

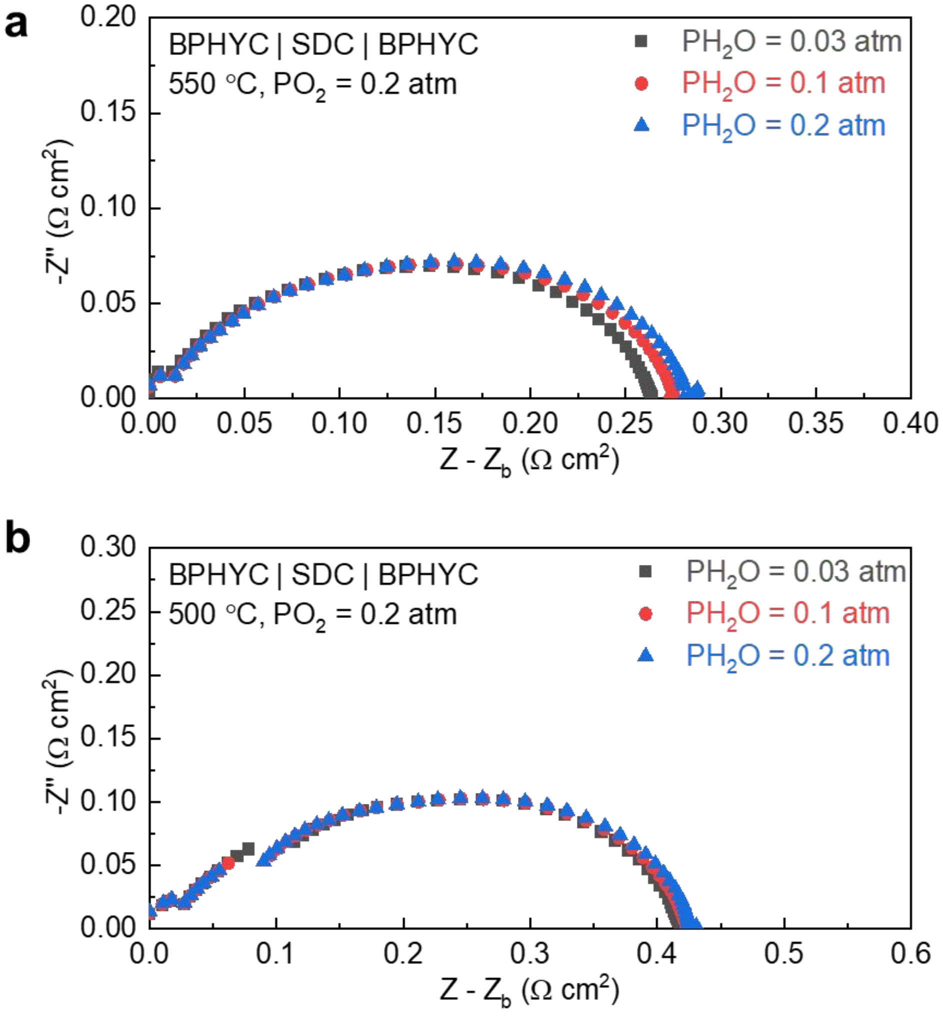

Figure S16. At PO_2 = 0.2 atm, EIS plots of BPHYC|SDC|BPHYC symmetrical cells at different concentrations of water at 550 °C (a) and 500 °C (b). When applied to oxygen-ion conducting electrolyte, increasing the steam concentration does not decrease the R_p of the symmetrical cell.

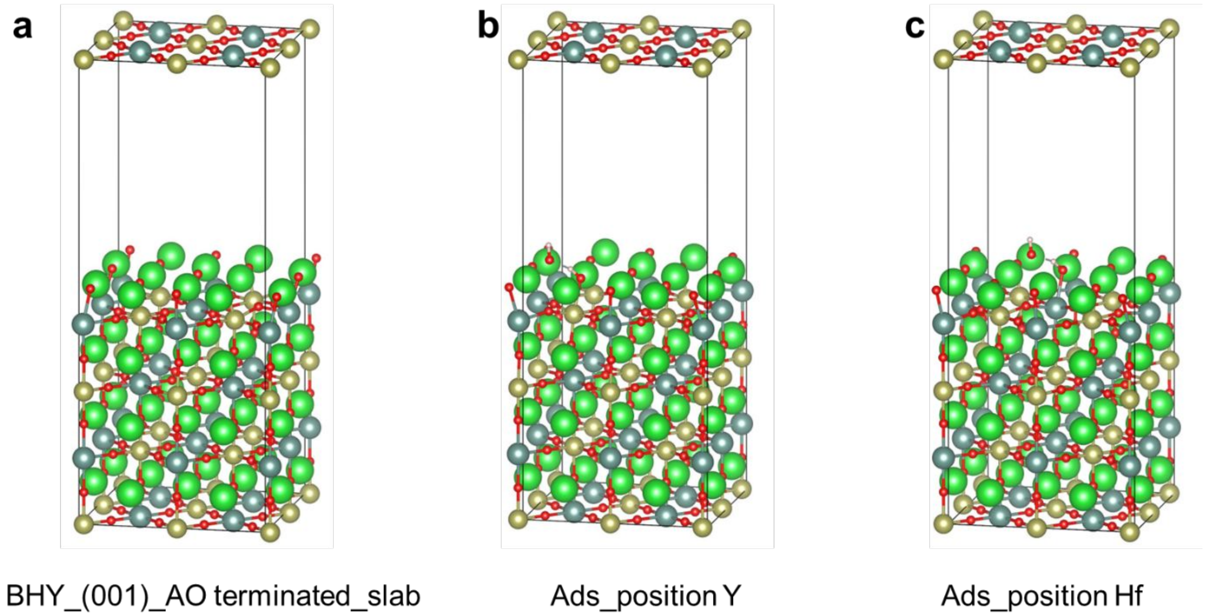

Figure S17. Water adsorption on BHY surface. (a) Slab model of BHY surface; (b) adsorption of water on Y site; (c) adsorption of water on Hf site.

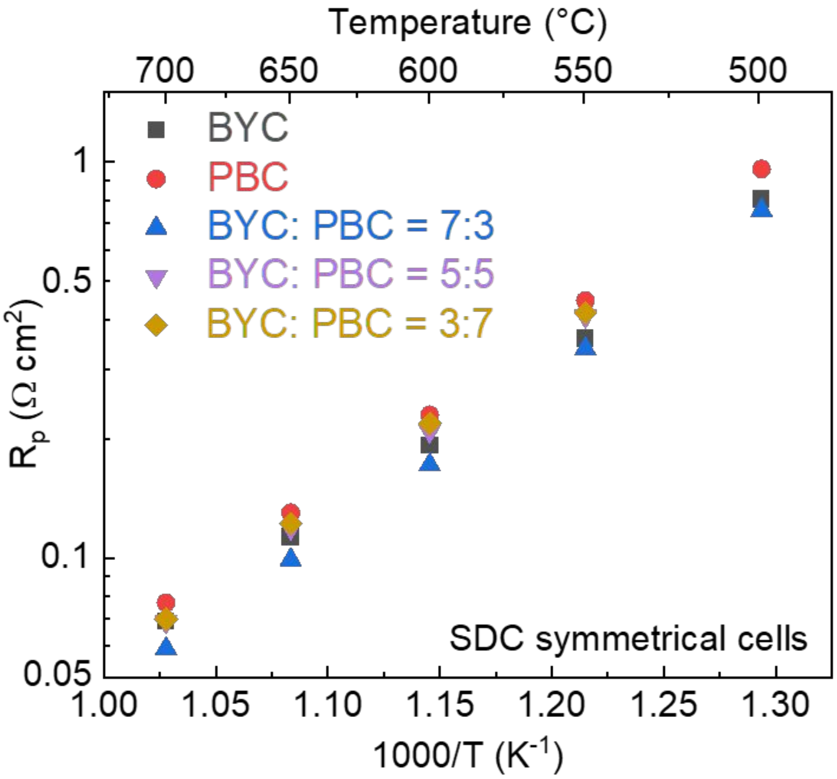

Figure S18. R_p of BYC and PBC at different mass ratios on SDC-based symmetrical cells.

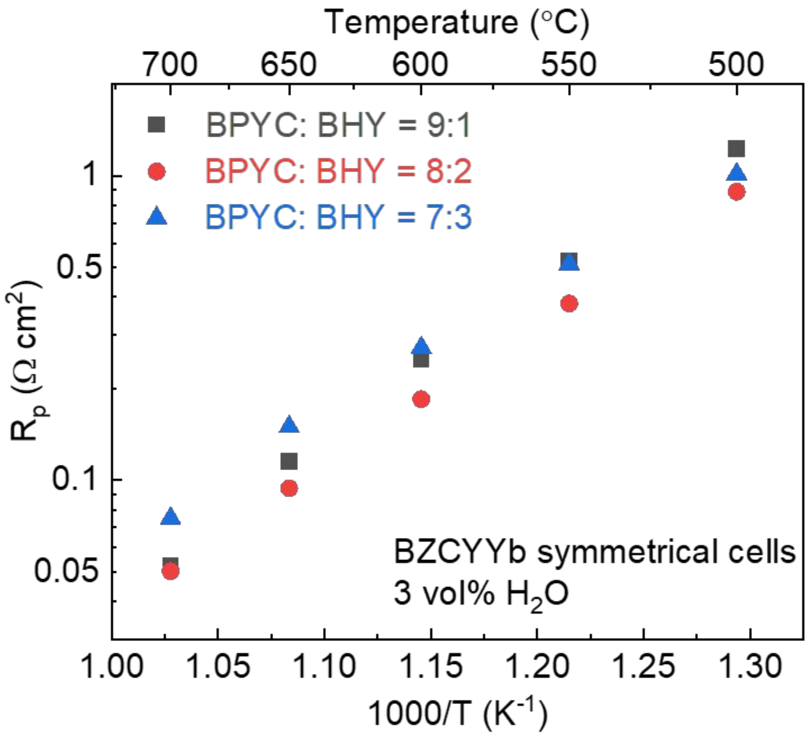

Figure S19. R_p of BPYC and BHY at different mass ratios on BZCYYb-based symmetrical cells. The optimized ratio is close to the one in BPHYC composite.

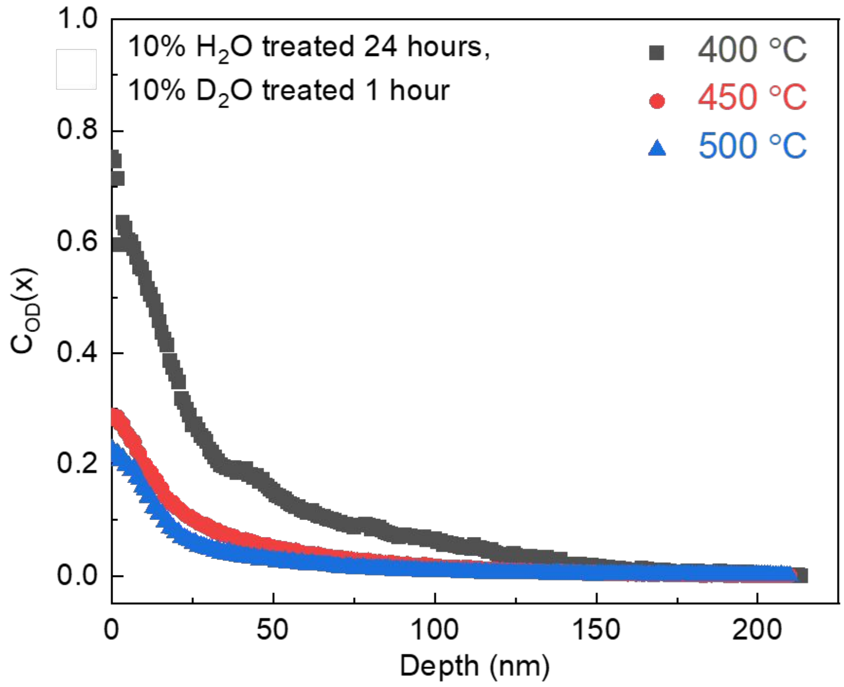

Figure S20. Normalized OD concentration profile as a function of the depth in the BPHYC sample.

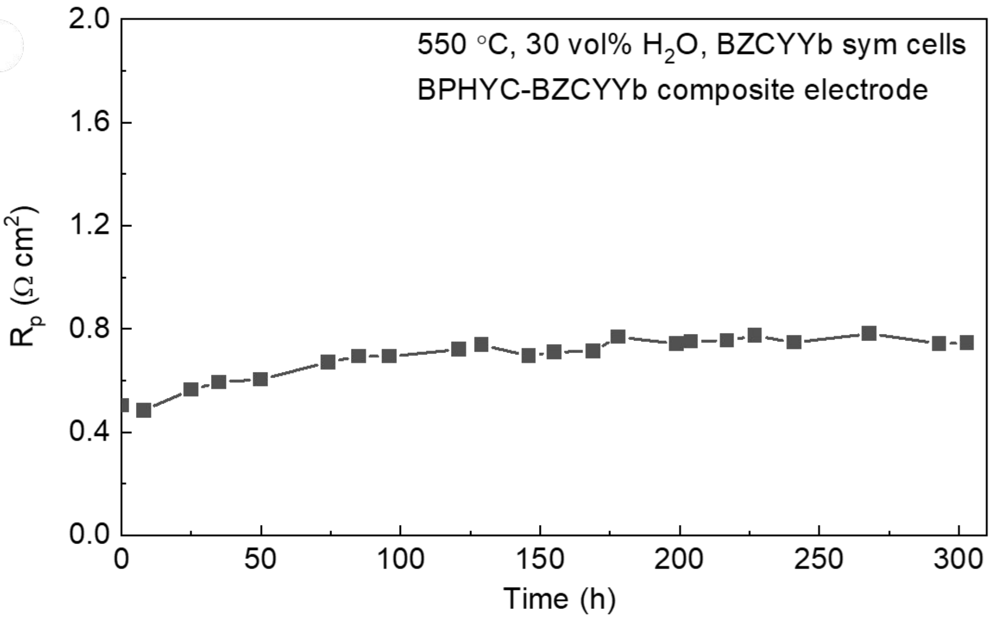

Figure S21. Electrochemical stability of BPHYC in 30 vol% H_2O on BZCYYb-based symmetrical cells. After initial performance degradation during the first 100 hours, the R_p of BPHYC-BZCYYb composite electrode remain stable for the rest of the 200 hours.

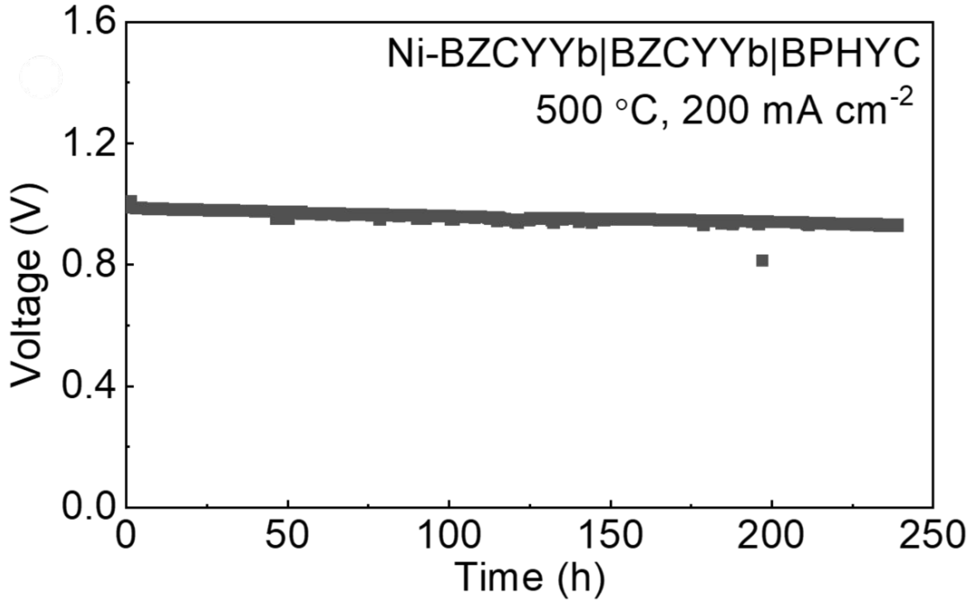

Figure S22. Stability of a single cell operated in the fuel cell mode at 500 °C for over 200 hours. Fuel electrode atmosphere: 20 sccm 3% H_2O-H_2, air electrode atmosphere: ambient air.

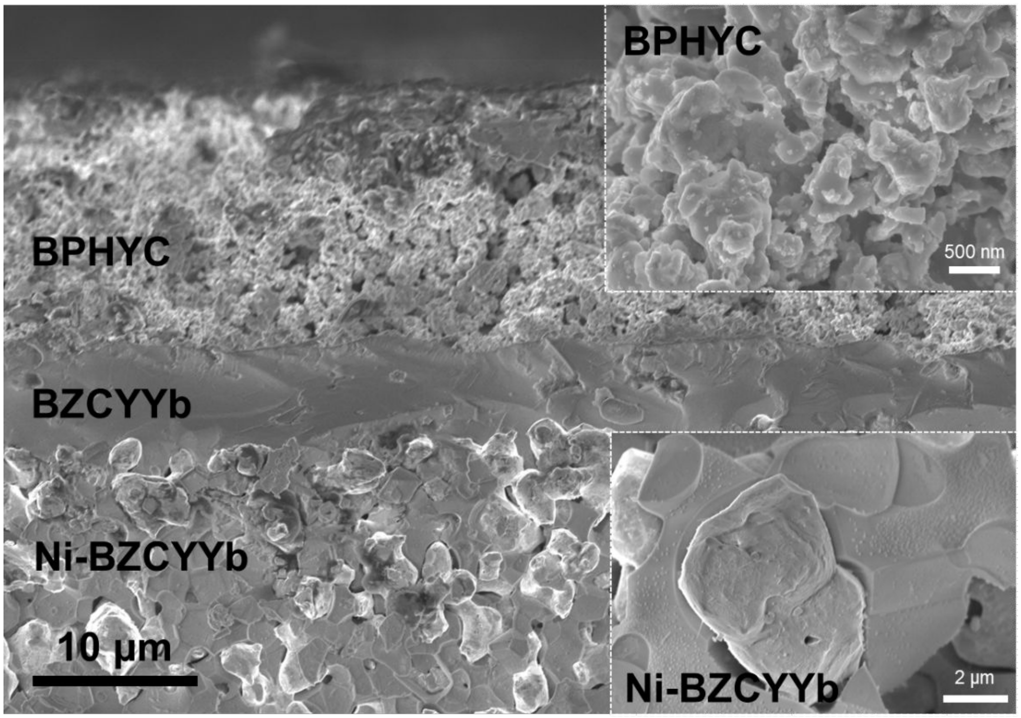

Figure S23. Cross-sectional view of the Ni-BZCYYb|BZCYYb|BPHYC single cell after testing in the electrolysis mode in 30 vol% H_2O for 300 hours (Figure 5h).

Table S1. Composition of the air electrode candidates

Material abbreviation	A-site cations (in mol%)		B-site cations (in mol%)				Composition
	Ba	Pr	Hf	Y	Co	Fe	
**BHYC4F4**	100	0	10	10	40	40	$BaHf_{0.1}Y_{0.1}Co_{0.4}Fe_{0.4}O_{3-\delta}$
**BHYC6F2**	100	0	10	10	60	20	$BaHf_{0.1}Y_{0.1}Co_{0.6}Fe_{0.2}O_{3-\delta}$
**BHYC**	100	0	10	10	80	0	$BaHf_{0.1}Y_{0.1}Co_{0.8}O_{3-\delta}$
**BPHYC**	90	10	10	10	80	0	$Ba_{0.9}Pr_{0.1}Hf_{0.1}Y_{0.1}Co_{0.8}O_{3-\delta}$
**BP2HYC**	80	20	10	10	80	0	$Ba_{0.8}Pr_{0.2}Hf_{0.1}Y_{0.1}Co_{0.8}O_{3-\delta}$
**BPH2YC**	90	10	20	10	70	0	$Ba_{0.9}Pr_{0.1}Hf_{0.2}Y_{0.1}Co_{0.7}O_{3-\delta}$
**BPHY2C**	90	10	10	20	70	0	$Ba_{0.9}Pr_{0.1}Hf_{0.1}Y_{0.2}Co_{0.7}O_{3-\delta}$
**BPH2Y2C**	90	10	20	20	60	0	$Ba_{0.9}Pr_{0.1}Hf_{0.2}Y_{0.2}Co_{0.6}O_{3-\delta}$

Table S2. Detailed information about the three phases in BPHYC

	Approximate composition	a (Å)	b (Å)	c (Å)	Mass fraction (%)
**BYC (phase A)**	$BaY_{0.15}Co_{0.85}O_{3-\delta}$	4.111	4.111	4.111	70.45
**PBC (phase B)**	$PrBaCo_2O_{5+\delta}$	3.905	3.905	7.618	10.95
**BHY (phase C)**	$BaHf_{0.9}Y_{0.1}O_{3-\delta}$	4.178	4.178	4.178	18.59

Table S3. Detailed information about the two phases in BPYC

	Approximate composition	a (Å)	b (Å)	c (Å)	Mass fraction (%)
**BYC (phase A)**	$BaY_{0.15}Co_{0.85}O_{3-\delta}$	4.096	4.096	4.096	85.53
**PBC (phase B)**	$PrBaCo_2O_{5+\delta}$	3.904	3.904	7.624	14.47

Table S4. Water adsorption energy on BHY surface

Material/energy (eV)	Slab energy	Position Y	Position Hf
BHY	-1272.77	-1288.47	-1288.42
Adsorption energy		-1.48	-1.43

Table S5. Water adsorption energy on BZCYYb surface

Material/energy (eV)	Slab energy	Position Zr	Position Yb	Position Y	Position Ce1	Position Ce2	Position Ce3
BZCYYb1711	-1212.43	-1228.29	-1228.34	-1228.36	-1228.22	-1228.09	-1228.06
Adsorption energy		-1.632	-1.685	-1.705	-1.568	-1.437	-1.407

REFERENCES

(1) Zhang, W.; Zhou, Y.; Liu, E.; Ding, Y.; Luo, Z.; Li, T.; Kane, N.; Zhao, B.; Niu, Y.; Liu, Y.; et al. A Highly Efficient and Durable Air Electrode for Intermediate-temperature Reversible Solid Oxide Cells. *Applied Catalysis B: Environmental* **2021**, *299*, 120631

(2) Zhou, Y.; Liu, E.; Chen, Y.; Liu, Y.; Zhang, L.; Zhang, W.; Luo, Z.; Kane, N.; Zhao, B.; Soule, L.; et al. An Active and Robust Air Electrode for Reversible Protonic Ceramic Electrochemical Cells. *ACS Energy Letters* **2021**, *6*, 1511-1520.

(3) Seong, A.; Kim, J.; Jeong, D.; Sengodan, S.; Liu, M.; Choi, S.; Kim, G. Electrokinetic Proton Transport in Triple ($H^+/O^{2-}/e^-$) Conducting Oxides as a Key Descriptor for Highly Efficient Protonic Ceramic Fuel Cells. *Advanced Science* **2021**, 2004099.

(4) Blochl, P. E. Projector Augmented-Wave Method. *Phys Rev B* **1994**, *50* (24), 17953-17979.

(5) Kresse, G.; Furthmuller, J. Efficiency of ab-initio total energy calculations for metals and semiconductors using a plane-wave basis set. *Comp Mater Sci* **1996**, *6* (1), 15-50.

(6) Cao, Y.; Gadre, M. J.; Ngo, A. T.; Adler, S. B.; Morgan, D. D. Factors controlling surface oxygen exchange in oxides. *Nat Commun* **2019**, *10* (1), 1346.

(7) Hu, X. Y.; Xie, Y.; Wan, Y. H.; Yang, Y.; Wu, X. J.; Xia, C. R. Antimony-doped strontium cobalt oxide as promising cathode for low-temperature solid oxide fuel cell with excellent carbon dioxide tolerance. *Appl Catal B-Environ* **2021**, *286*, 119901.

(8) Cao, Y. P.; Gadre, M. J.; Ngo, A. T.; Adler, S. B.; Morgan, D. D. Factors controlling surface oxygen exchange in oxides. *Nat Commun* **2019**, *10*, 1346.

(9) Henkelman, G.; Uberuaga, B. P.; Jonsson, H. A climbing image nudged elastic band method for finding saddle points and minimum energy paths. *J Chem Phys* **2000**, *113* (22), 9901-9904.

(10) Zhang, W. L.; Hu, X. Y.; Zhou, Y. C.; Luo, Z. Y.; Nam, G.; Ding, Y.; Li, T. T.; Liu, Z. J.; Ahn, Y. J.; Kane, N.; et al. A Solid Oxide Fuel Cell Runs on Hydrocarbon Fuels with Exceptional Durability and Power Output. *Adv Energy Mater* **2022**, *12* (47), 2202928.

(11) Luo, Z. Y.; Zhou, Y. C.; Hu, X. Y.; Kane, N.; Zhang, W. L.; Li, T. T.; Ding, Y.; Liu, Y.; Liu, M. L. Highly Conductive and Durable Nb(Ta)-Doped Proton Conductors for Reversible Solid Oxide Cells. *Acs Energy Lett* **2022**, 2970-2978.
